# Supplementary material for: Field testing of an enzymatic quorum quencher coating additive to reduce biocorrosion of steel
Source: Microbiol Spectr. 2023 Sep 5;11(5):e05178-22. doi: 10.1128/spectrum.05178-22 (PMC10580884; doi:10.1128/spectrum.05178-22)
Supplement: Supplemental file 1 — Supplemental material. [file spectrum.05178-22-s0001.docx]

Supporting Information

Field testing of an enzymatic quorum quencher coating additive to reduce biocorrosion of steel

Siqian Huang^1,2^, Celine Bergonzi^2‡^, Sherry Smith^3^, Randall E. Hicks^1^, Mikael H. Elias^2^

^1^University of Minnesota Duluth, Department of Biology, Duluth, MN, USA

^2^University of Minnesota, Department of Biochemistry, Molecular Biology and Biophysics & Biotechnology Institute, St. Paul, MN, USA

^3^Independant Scholar, Minneapolis, MN, USA.

^‡^ Present address: **Univ. Grenoble Alpes, CNRS, CEA, IBS, F-38000 Grenoble, France.**

Summary

[**Table S1**. Analysis of molecular variance (AMOVA) showing the genetic variation among different treatment groups, sampling times, sites, and depths. 4](#_Toc134010067)

[**Table S2**. Summary of potential AHL-producing, AHL-degrading, and AHL-sensing ability of the bacterial orders of highest relative abundance. 6](#_Toc134010068)

[**Fig S1**. Schematic of a coating-treated steel coupon and the scratches. 7](#_Toc134010069)

[**Fig S2**. SsoPox lactonase enzymatic activity in the acrylic coating and the silica gel coating. 8](#_Toc134010070)

[**Fig S3**. Photographs of control and treated steel coupons after 1, 2, 8 and 21 months of exposure in the Duluth-Superior Harbor (Site: HD5, Depth: 3m). 10](#_Toc134010071)

[**Fig S4**. Counts of corrosion tubercles on the duplicate unscratched and scratched steel coupons with different experimental treatments at the two tested sites (HD5 and LAF) and depths (1 and 3m). 12](#_Toc134010072)

[**Fig S5**. Tubercle counts (upper panel) and coverage (lower panel) of corrosion tubercles on bare steel coupons at 4 sampling times. 13](#_Toc134010073)

[**Fig S6**. Mussel counts on unscratched steel coupons from different experimental treatments. 14](#_Toc134010074)

[**Fig. S7**. Mussel weights (g) on unscratched steel coupons from different experimental treatments. 15](#_Toc134010075)

[**Fig S8**. Mussel counts on scratched steel coupons from different experimental treatments. 16](#_Toc134010076)

[**Fig S9**. Mussel weights (g) on scratched steel coupons from different experimental treatments. 17](#_Toc134010077)

[**Fig S10**. Heatmap comparing the relative abundance of partial 16S rRNA sequences and OTU richness for the top 40 bacterial orders in all samples from each treatment and control. 18](#_Toc134010078)

[**Fig S11**. Relative abundance of partial 16S rRNA sequences for the top 40 bacterial orders of all samples from each treatment and control. 20](#_Toc134010079)

[**Fig S12**. Nonmetric multidimensional scaling plot showing the differences between bacterial communities in different treatments on corroding steel coupons grouped by site and sample exposure time. 21](#_Toc134010080)

[**Fig S13**. Nonmetric multidimensional scaling plot showing the differences between bacterial communities in different treatments on corroding steel coupons over time. 22](#_Toc134010081)

[**Fig S14**. Nonmetric multidimensional scaling plot showing the differences between bacterial communities in different treatments on corroding steel coupons grouped by site and sample exposure time. The site, depth and months of each plot is shown above the graph. The 4 treatment groups are separated by different colors. 23](#_Toc134010082)

[**Fig S15**. Average iron-oxidizing and sulfate-reducing bacteria relative abundances on steel coupons with different experimental treatments. 24](#_Toc134010083)

[**Fig S16**. Log scale plots showing the correlation between the relative abundance of bacterial orders and the tubercle coverage for the 40 most abundant bacterial orders by total sequence counts for treatment and control samples. 27](#_Toc134010084)

[**Fig S17**. The linear discriminant analysis (LDA) effect size (LEfSe) analysis identified bacterial orders that responded significantly to the different treatments during the four sampling periods. 29](#_Toc134010085)

[References for Table S2: 30](#_Toc134010086)

# **Table S1**. Analysis of molecular variance (AMOVA) showing the genetic variation among different treatment groups, sampling times, sites, and depths.

| Compared parameter | Compared samples | AMOVA FST (%) | p-value |
| --- | --- | --- | --- |
| Treatments | **Acrylic_Control-Bare_Steel_Control-all samples** | 2.92137 | 0.003* |
|  | **Acrylic_Control-Lactonase-all samples**  1 month-HD5-1m  1 month-HD5-3m  1 month-LAF-1m  1 month-LAF-3m  2 months-HD5-1m  2 months-HD5-3m  2 months-LAF-1m  2 months-LAF-3m  8 months-HD5-1m  8 months-HD5-3m  8 months-LAF-1m  8 months-LAF-3m  21 months-HD5-1m  21 months-HD5-3m  21 months-LAF-1m  21 months-LAF-3m | 1.50319  3.45146  1.23856  1.86708  0.78288  1.33684  0.71462  0.31260  1.55857  2.14143  2.20429  2.07265  3.37534  4.66278  3.29742  3.62930  4.24146 | 0.213  0.164  0.436  0.134  0.335  0.147  0.45  0.733  0.214  0.281  0.368  0.312  0.202  0.105  0.066  0.128  0.074 |
|  | **Acrylic_Control-Surfactin-all samples**  1 month-HD5-1m  1 month-HD5-3m  1 month-LAF-1m  1 month-LAF-3m  2 months-HD5-1m  2 months-HD5-3m  2 months-LAF-1m  2 months-LAF-3m  8 months-HD5-1m  8 months-HD5-3m  8 months-LAF-1m  8 months-LAF-3m  21 months-HD5-1m  21 months-HD5-3m  21 months-LAF-1m  21 months-LAF-3m | 1.02473  3.10409  1.18736  1.92413  2.14667  0.41522  0.62950  0.25412  2.05739  2.57217  2.97855  3.47653  3.36844  2.96327  1.83590  5.96721  3.85772 | 0.409  0.188  0.496  0.142  0.125  0.526  0.689  0.740  0.196  0.263  0.185  0.156  0.180  0.206  0.178  0.055  0.096 |
|  | **Bare_Steel_Control-Lactonase-all samples** | 5.87454 | <0.001* |
|  | **Bare_Steel_Control-Surfactin-all samples** | 4.23742 | <0.001* |
|  | **Lactonase-Surfactin-all samples**  1 month-HD5-1m  1 month-HD5-3m  1 month-LAF-1m  1 month-LAF-3m  2 months-HD5-1m  2 months-HD5-3m  2 months-LAF-1m  2 months-LAF-3m  8 months-HD5-1m  8 months-HD5-3m  8 months-LAF-1m  8 months-LAF-3m  21 months-HD5-1m  21 months-HD5-3m  21 months-LAF-1m  21 months-LAF-3m | 0.95457  0.13241  0.21687  0.24552  0.89360  1.21166  0.46538  0.28542  1.26277  1.89201  2.54260  2.18812  1.02574  3.17190  2.91945  6.41289  0.96621 | 0.467  0.872  0.769  0.624  0.335  0.217  0.640  0.795  0.266  0.311  0.283  0.188  0.213  0.107  0.084  0.053  0.642 |
| Exposure Period | 1 Month – 2 Months | 59.2133 | <0.001* |
|  | 1 Month - 21 Months | 32.8885 | <0.001* |
|  | 1 Month - 8 Months | 35.0827 | <0.001* |
|  | 2 Months -21 Months | 47.9193 | <0.001* |
|  | 2 Months -8 Months | 54.6192 | <0.001* |
|  | 21 Months -8 Months | 3.22321 | 0.007* |
| Site | HD5-LAF | 2.42668 | 0.02* |
| Depth | 1 M-3 M | 5.5809 | <0.001* |

# **Table S2**. Summary of potential AHL-producing, AHL-degrading, and AHL-sensing ability of the bacterial orders of highest relative abundance. The table was assembled by surveying the literature and identifying reports suggesting interaction with AHL for a bacteria member of the order.

| Orders of Highest Abundance | order Type | AHL Producing | AHL Degrading | AHL Sensing | References |
| --- | --- | --- | --- | --- | --- |
| *BDELLOVIBRIONALES* | SRB |  |  | * | [1] |
| *DESULFOVIBRIONALES* | SRB | * | * | * | [2-6] |
| *DESULFUROMONADALES* | SRB | * |  | * | [2, 7-8] |
| *METHYLOCOCCALES* | SRB | * |  | * | [9-10] |
| *METHYLOPHILALES* | SRB | * |  | * | [10] |
| *SULFURICELLALES* | SRB |  |  | * | [11] |
| *VERRUCOMICROBIALES* | SRB |  |  | * | [10,12] |
| *ACIDIMICROBIALES* | IOB | * | * | * | [10] |
| *ACTINOMYCETALES* | IOB | * | * | * | [13-14] |
| *BACTEROIDALES* | IOB |  |  | * | [12,15] |
| *BURKHOLDERIALES* | IOB | * |  | * | [10,12,16-17] |
| *FLAVOBACTERIALES* | IOB |  | * | * | [10,12,18] |
| *GALLIONELLALES* | IOB |  |  | * | [19] |
| *HOLOPHAGALES* | IOB |  |  | * | [12] |
| *NITROSOMONADALES* | IOB | * |  | * | [20-22] |
| *NITROSPIRALES* | IOB | * | * | * | [10, 19, 23] |
| *PLANCTOMYCETALES* | IOB |  |  | * | [24] |
| *RHIZOBIALES* | IOB | * | * | * | [10,18,25] |
| *RHODOBACTERALES* | IOB | * | * | * | [10,18,25-27] |
| *RHODOCYCLALES* | IOB |  | * | * | [12, 28] |
| *RHODOSPIRILLALES* | IOB | * | * | * | [10,29-30] |
| *SPHINGOBACTERIALES* | IOB | * | * | * | [10,18,31-32] |
| *SPHINGOMONADALES* | IOB | * | * | * | [33-34] |
| *ACIDOBACTERIA* | others | * |  | * | [23,35] |
| *ARIDIBACTER* | others |  |  |  | ǂ |
| *BACILLALES* | others | * | * | * | [36-38] |
| *CAULOBACTERALES* | others | * |  |  | [32] |
| *CYTOPHAGALES* | others |  | * |  | [10, 39] |
| *GAIELLALES* | others |  |  |  | ǂ |
| *GEMMATIMONADALES* | others |  | * |  | [10] |
| *MYXOCOCCALES* | others | * | * | * | [10,12,40-41] |
| *OPITUTALES* | others |  | * |  | [10] |
| *PSEUDOMONADALES* | others | * | * | * | [32, 42-44] |
| *RICKETTSIALES* | others | * |  | * | [45] |
| *SACCHARIBACTERIA* | others |  |  |  | ǂ |
| *XANTHOMONADALES* | others |  | * | * | [46-49] |

ǂ information could not be found in the literature.

11.4 cm

4.8 cm

Coated surface

Coated surface

3 mm width scratches

Coated surface

# **Fig S1**. Schematic of a coating-treated steel coupon and the scratches.

# **Fig S2**. SsoPox lactonase enzymatic activity in the acrylic coating and the silica gel coating. Values are normalized to the average absorbance reading value for the silica gel coating since this coating was used in our previous study (Huang et al., 2019). The enzyme activity was represented with the normalized absorbance at 412 nm.

1 month

2 months

D: 200 ug/ml lactonase

C: 200 ug/ml surfactin

B: Acrylic coating control

Unscratched Scratched

A: No coating

control

Unscratched Scratched

Unscratched Scratched


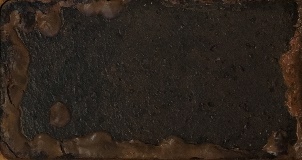

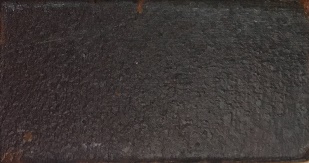

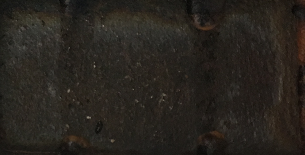

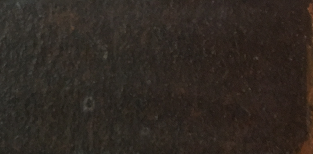

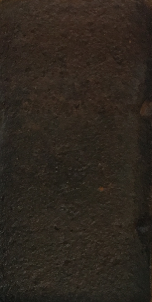

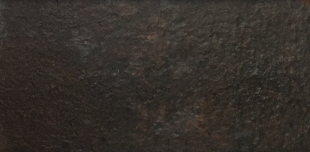

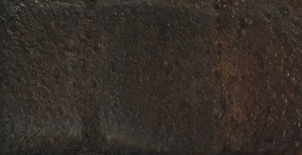

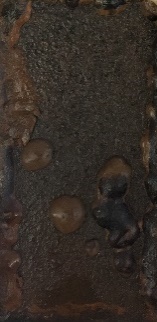

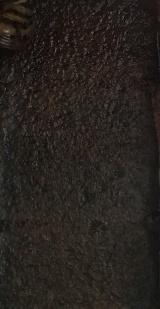

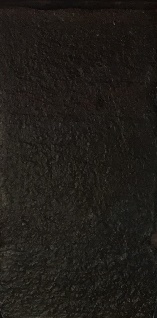

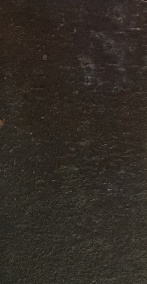

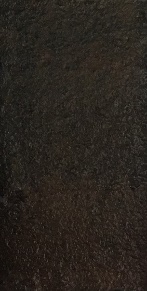

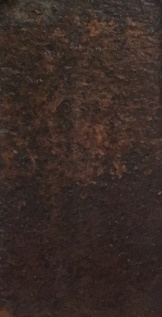

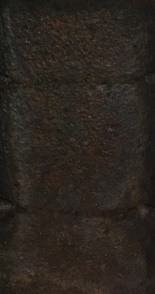


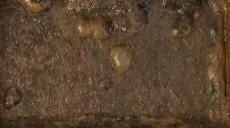

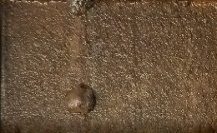

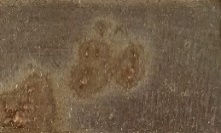

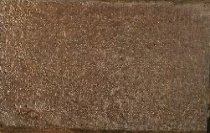

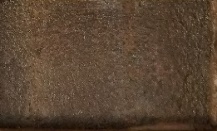

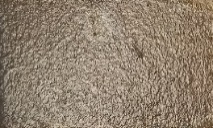

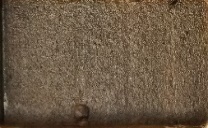


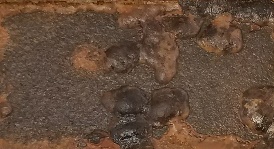

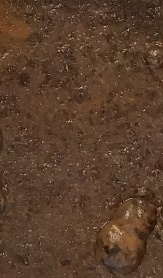

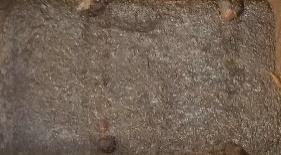

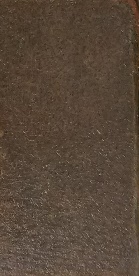

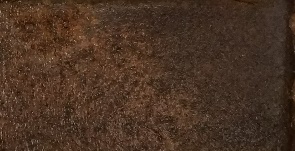

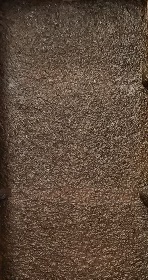

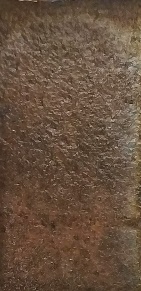

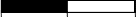


0 2 4cm

21 months

D: 200 ug/ml lactonase

C: 200 ug/ml surfactin

B: Acrylic coating control

Unscratched Scratched

A: No coating

control

Unscratched Scratched

Unscratched Scratched


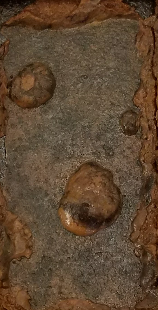

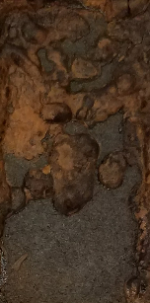

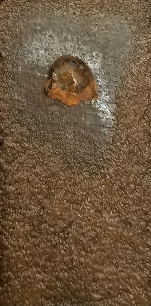

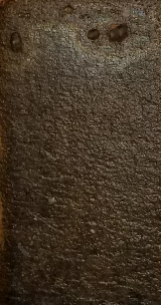

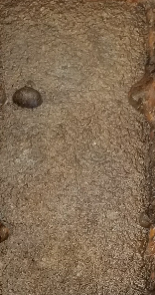

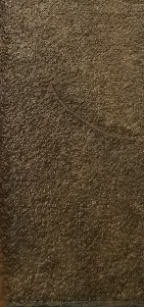

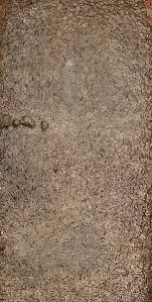


8 months


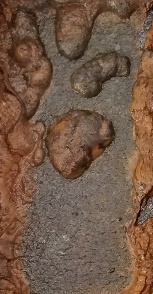

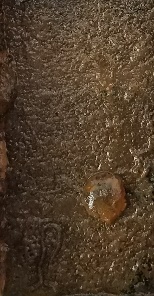

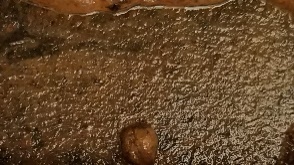

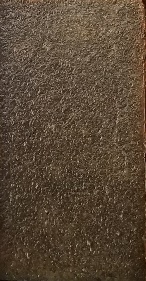

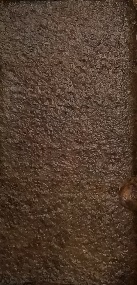

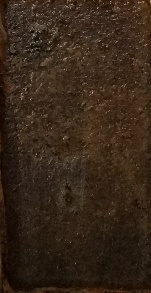

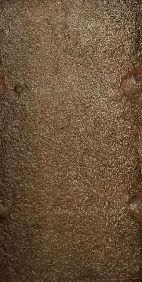


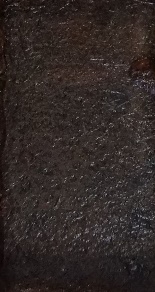

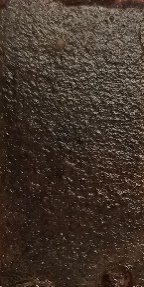

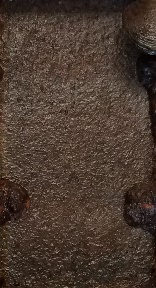

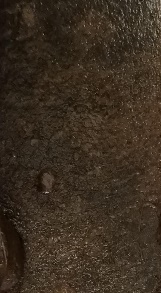

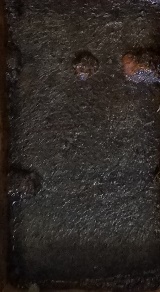

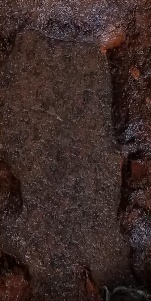

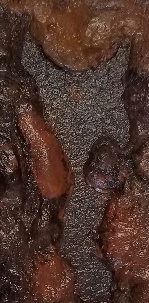


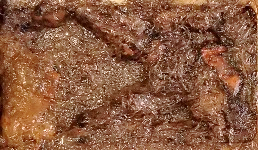

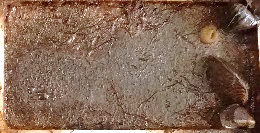

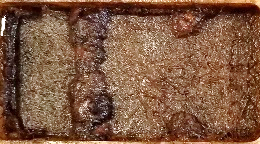

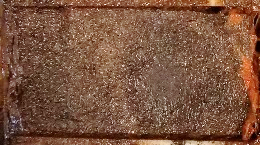

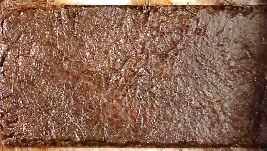

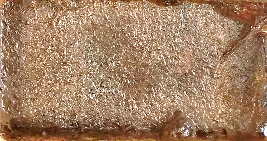

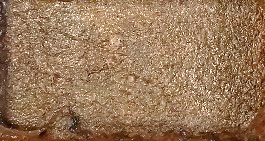

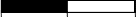


0 2 4cm

# **Fig S3**. Photographs of control and treated steel coupons after 1, 2, 8 and 21 months of exposure in the Duluth-Superior Harbor (Site: HD5, Depth: 3m). This set of pictures is representative of sample coupons at other sites/depth. The image on the left in each test group is the unscratched side of the coupon.


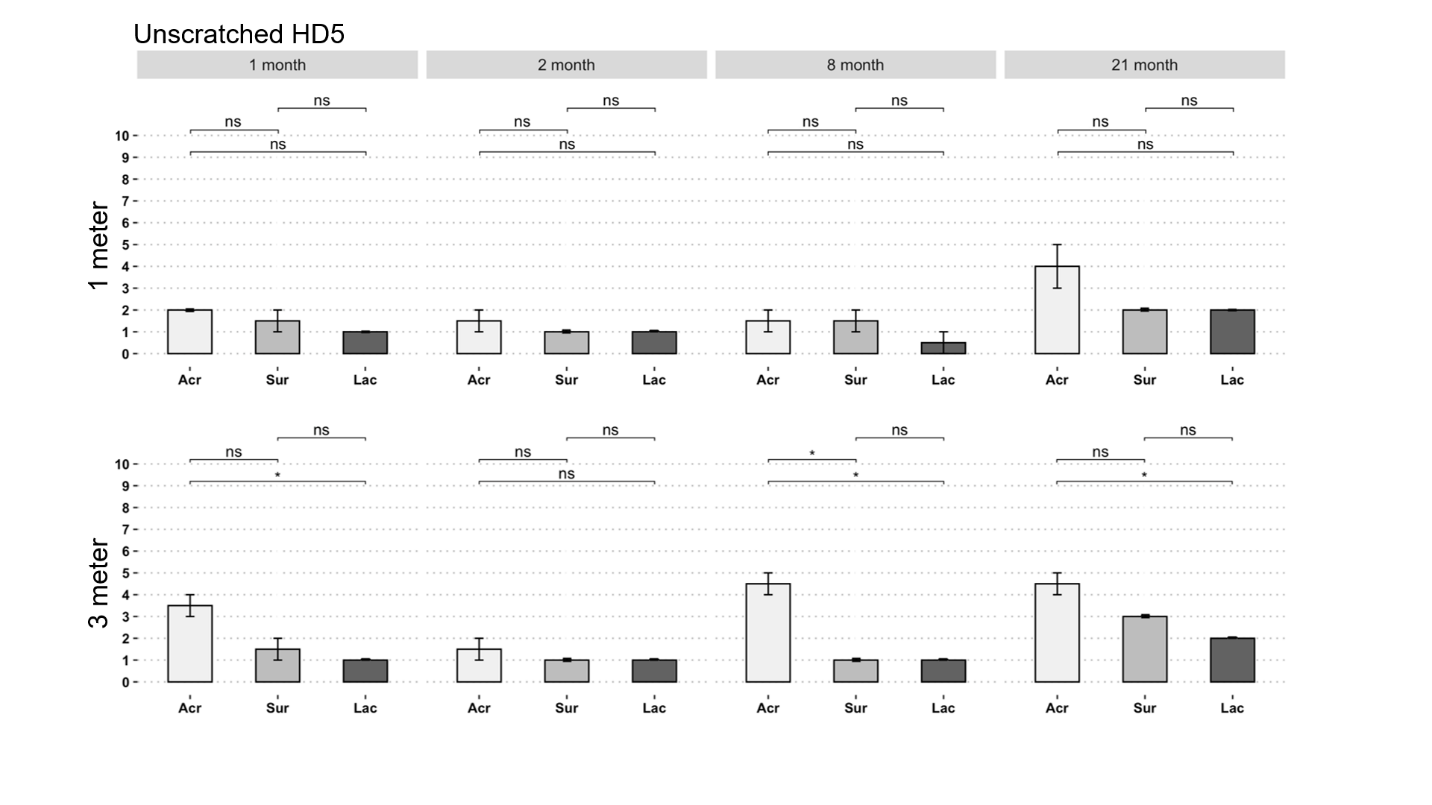

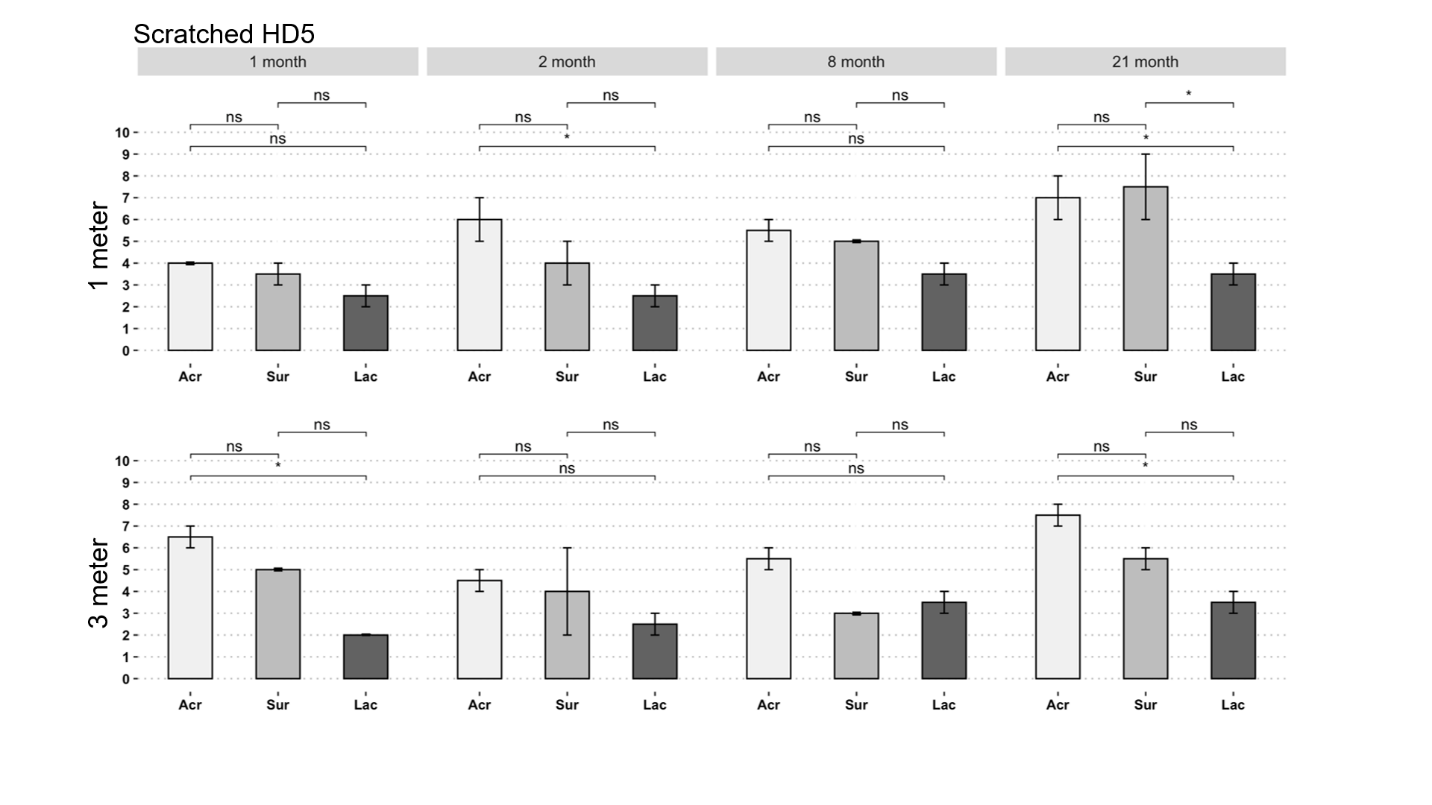


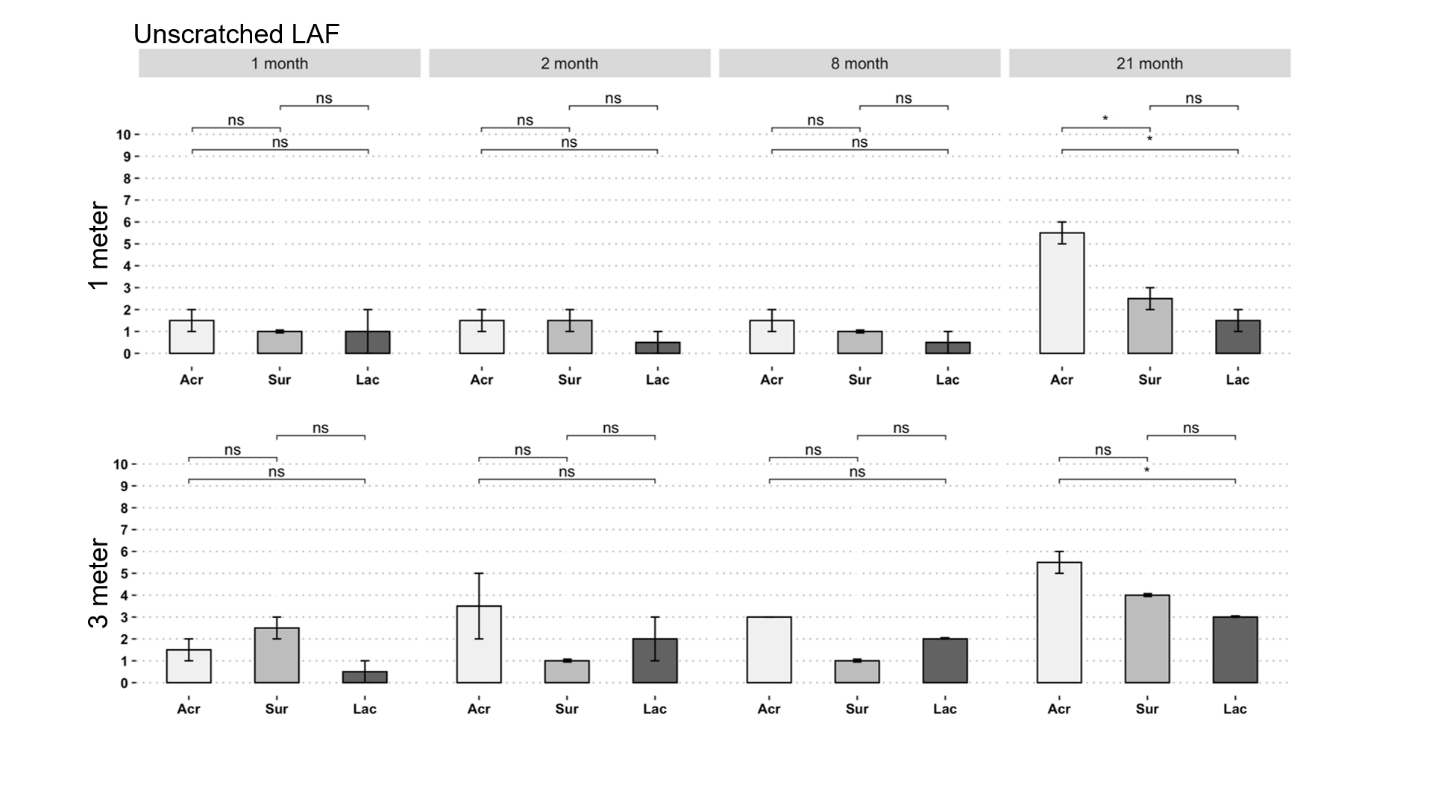

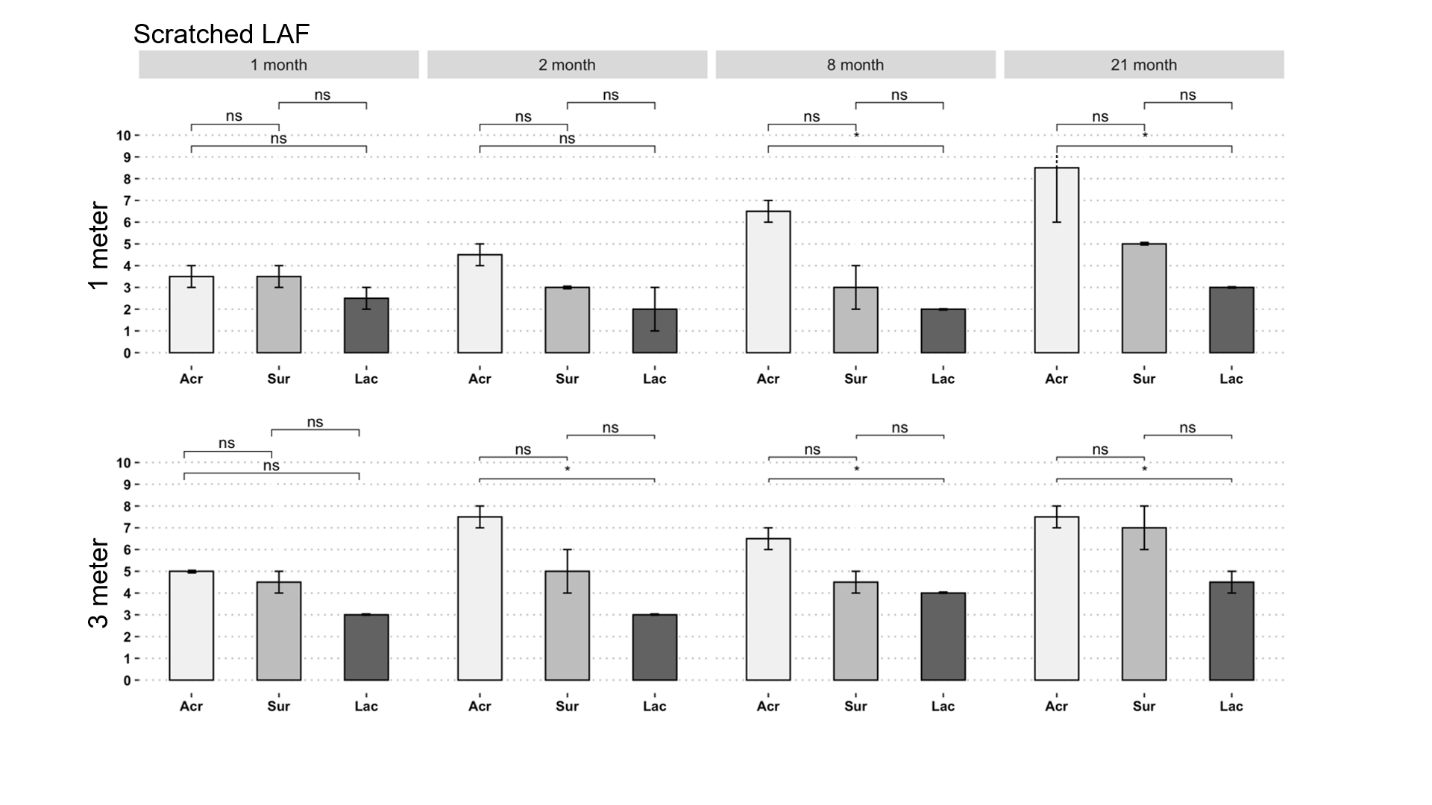


# **Fig S4**. Counts of corrosion tubercles on the duplicate unscratched and scratched steel coupons with different experimental treatments at the two tested sites (HD5 and LAF) and depths (1 and 3m). Legend - Acr: Acrylic coating control, Sur: Acrylic coating with 200 ug/ml surfactin, Lac: Acrylic coating with 200 ug/ml *Sso*Pox lactonase enzyme.

# **Fig S5**. Tubercle counts (upper panel) and coverage (lower panel) of corrosion tubercles on bare steel coupons at 4 sampling times. Mean values for 2 coupons at both sites are shown (n=4).


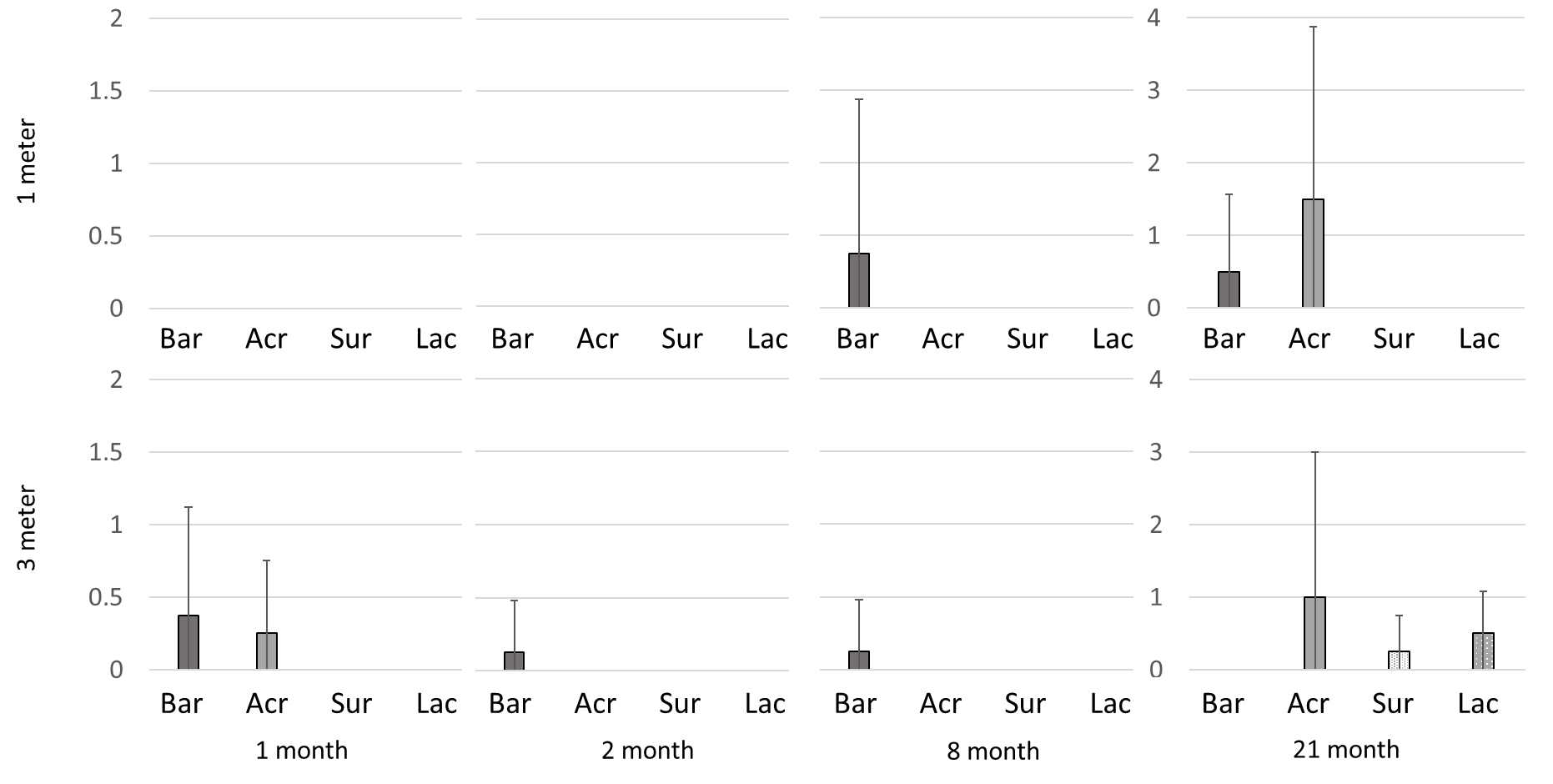


# **Fig S6**. Mussel counts on unscratched steel coupons from different experimental treatments. Mean values for 2 coupons at both sites are shown (n=4). Legend - Bar: Bare Steel, Acr: Acrylic coating control, Sur: Acrylic coating with 200 ug/ml surfactin, Lac: Acrylic coating with 200 ug/ml *Sso*Pox lactonase enzyme.


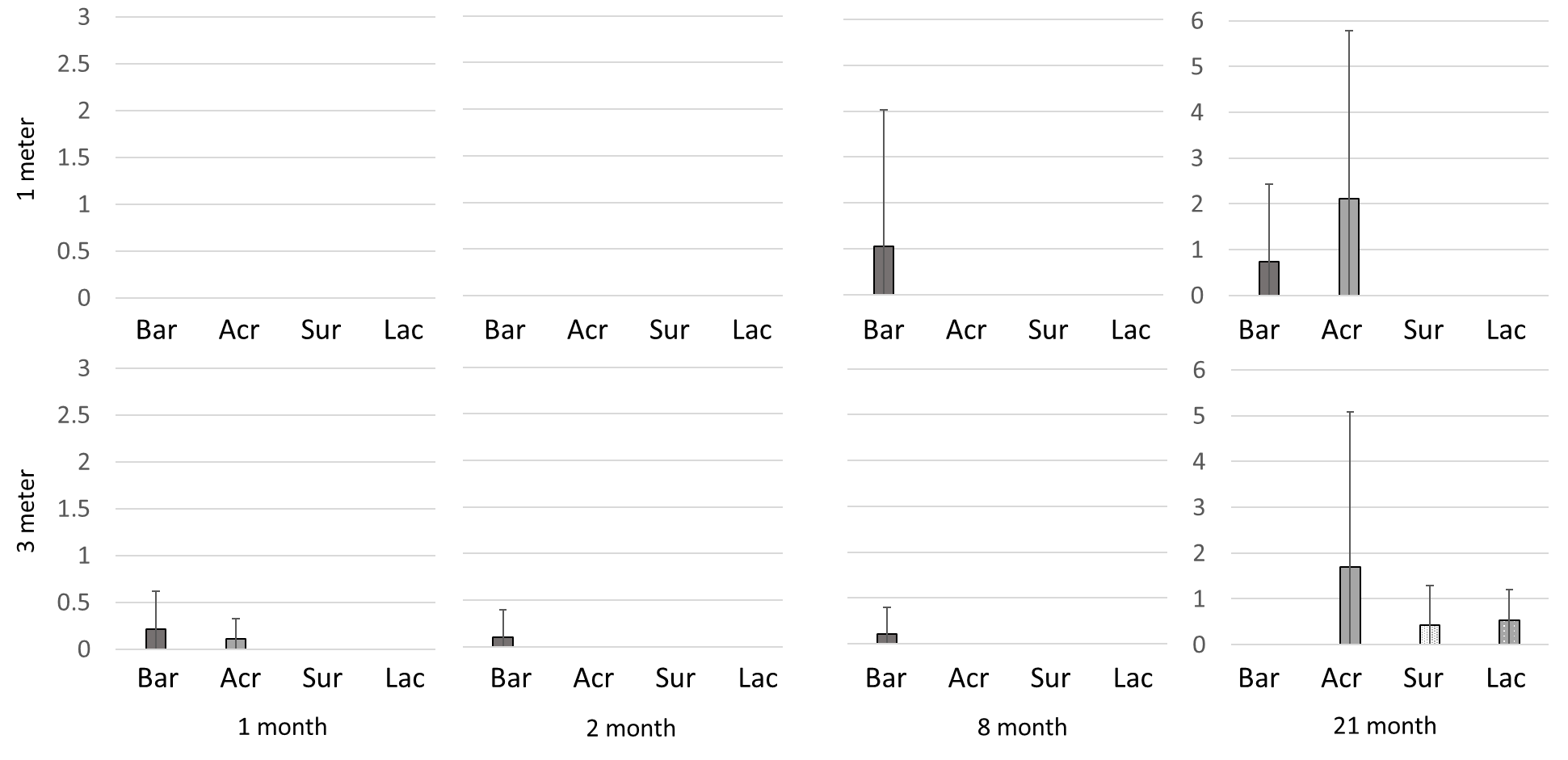


# **Fig. S7**. Mussel weights (g) on unscratched steel coupons from different experimental treatments. Mean values for 2 coupons at both sites are shown (n=4). Legend - Bar: Bare Steel, Acr: Acrylic coating control, Sur: Acrylic coating with 200 ug/ml surfactin, Lac: Acrylic coating with 200 ug/ml *Sso*Pox lactonase enzyme.


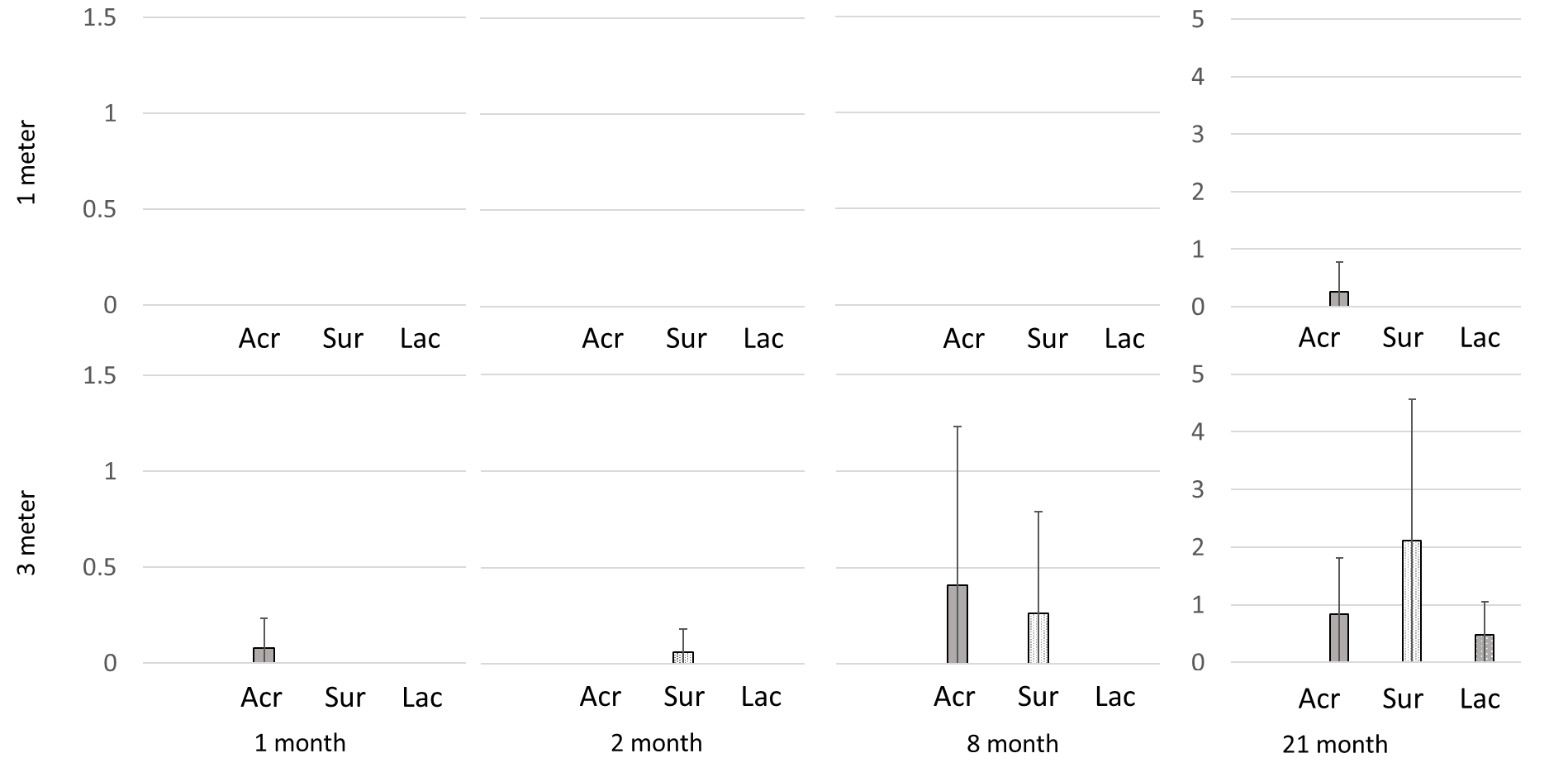


# **Fig S8**. Mussel counts on scratched steel coupons from different experimental treatments. Mean values for 2 coupons at both sites are shown (n=4). Legend - Acr: Acrylic coating control, Sur: Acrylic coating with 200 ug/ml surfactin, Lac: Acrylic coating with 200 ug/ml *Sso*Pox lactonase enzyme.


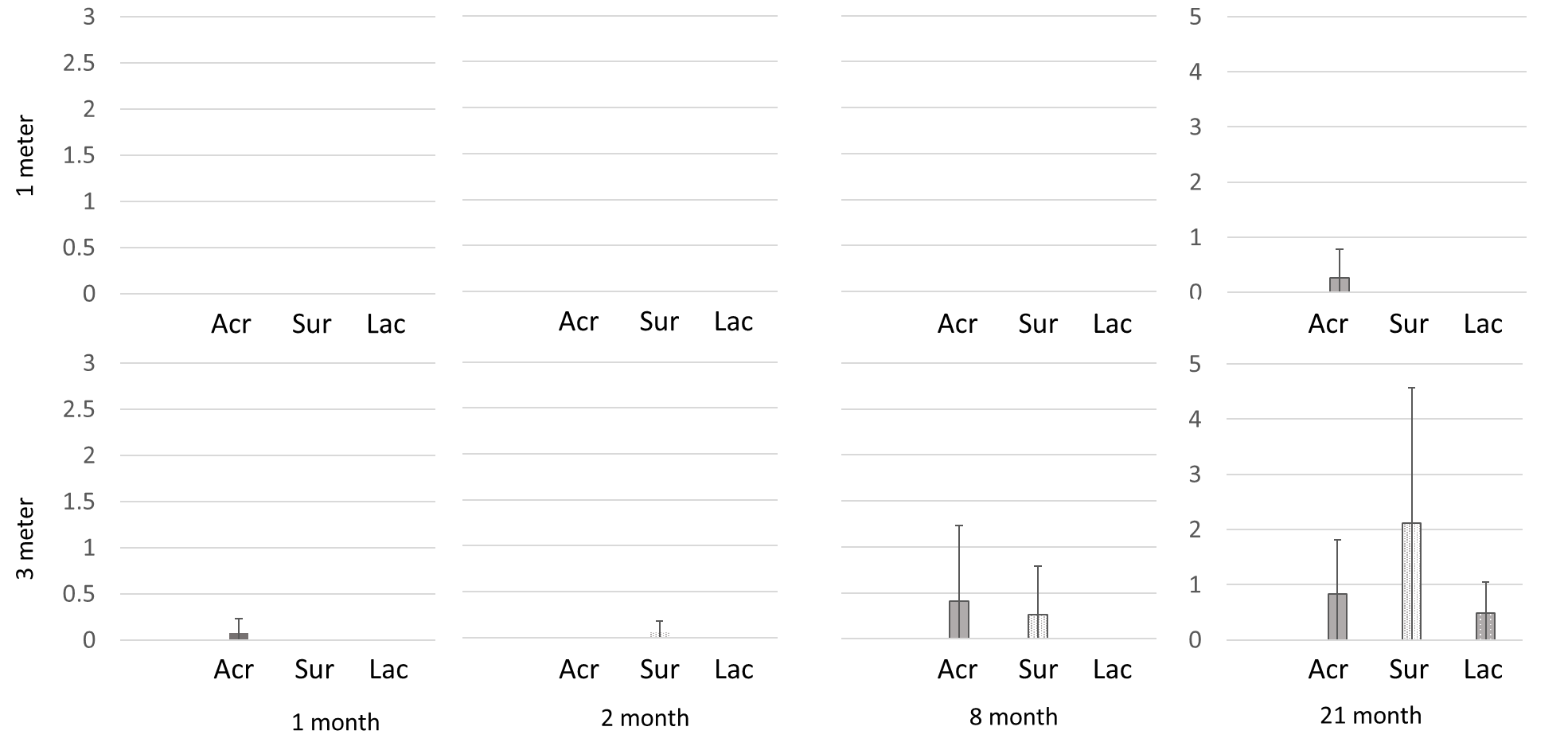


# **Fig S9**. Mussel weights (g) on scratched steel coupons from different experimental treatments. Mean values for 2 coupons at both sites are shown (n=4). Legend - Acr: Acrylic coating control, Sur: Acrylic coating with 200 ug/ml surfactin, Lac: Acrylic coating with 200 ug/ml *Sso*Pox lactonase enzyme.

# **
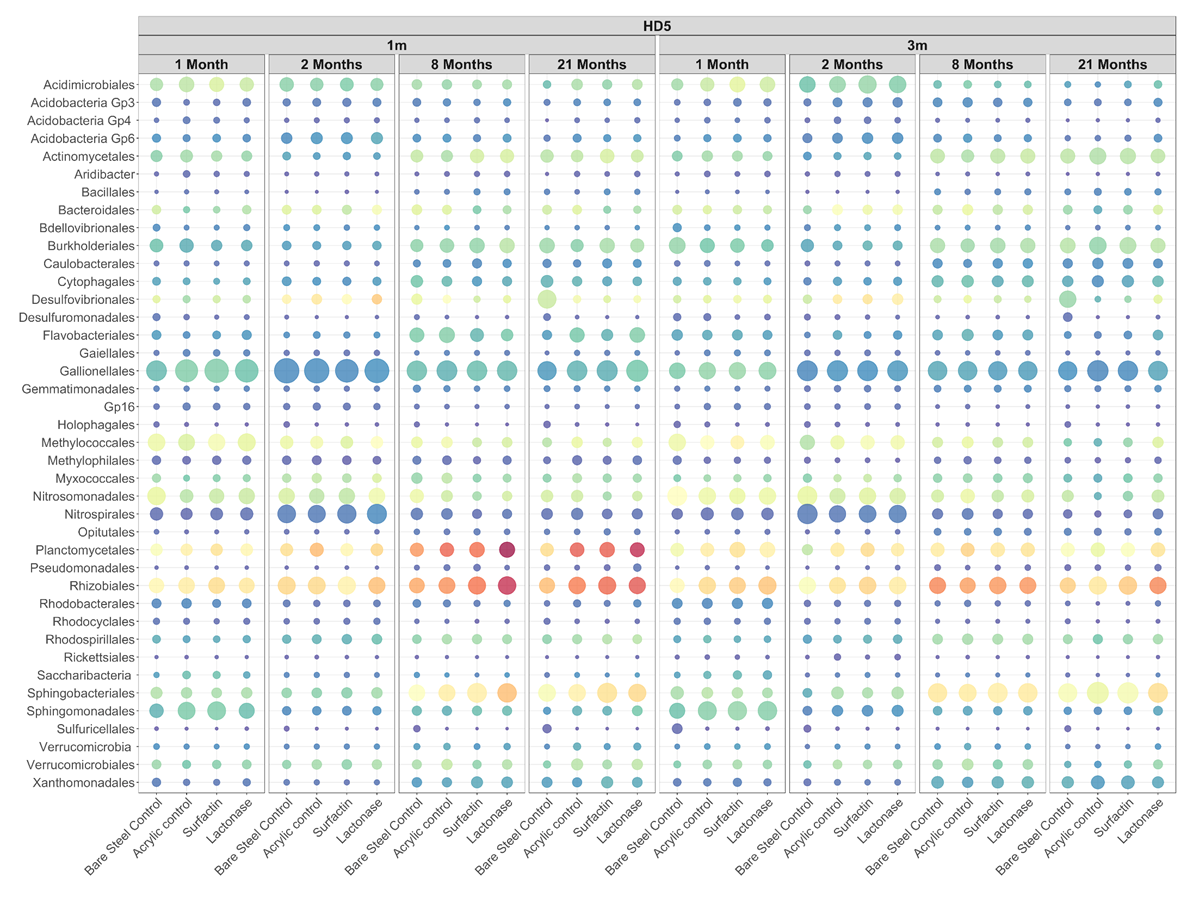

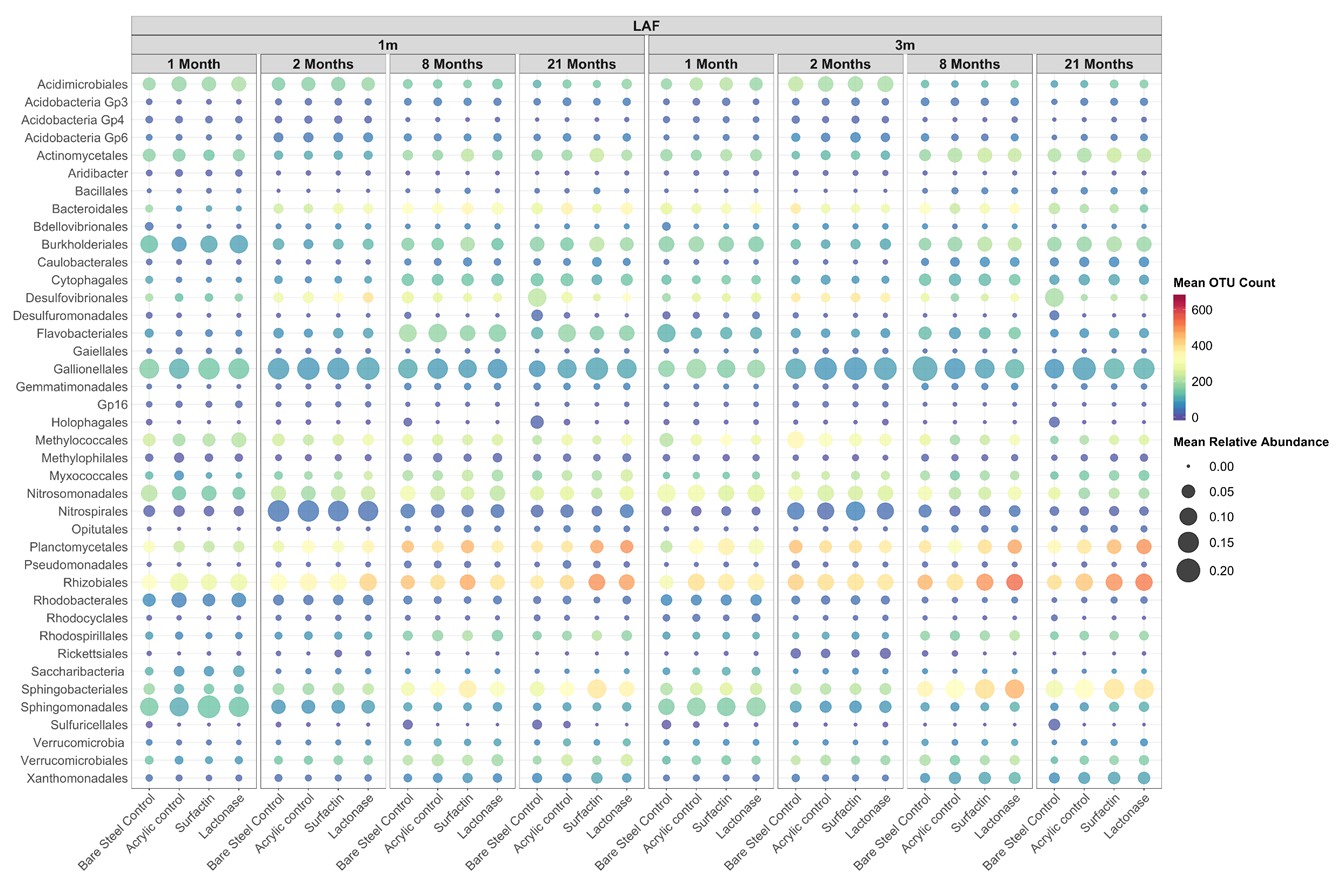

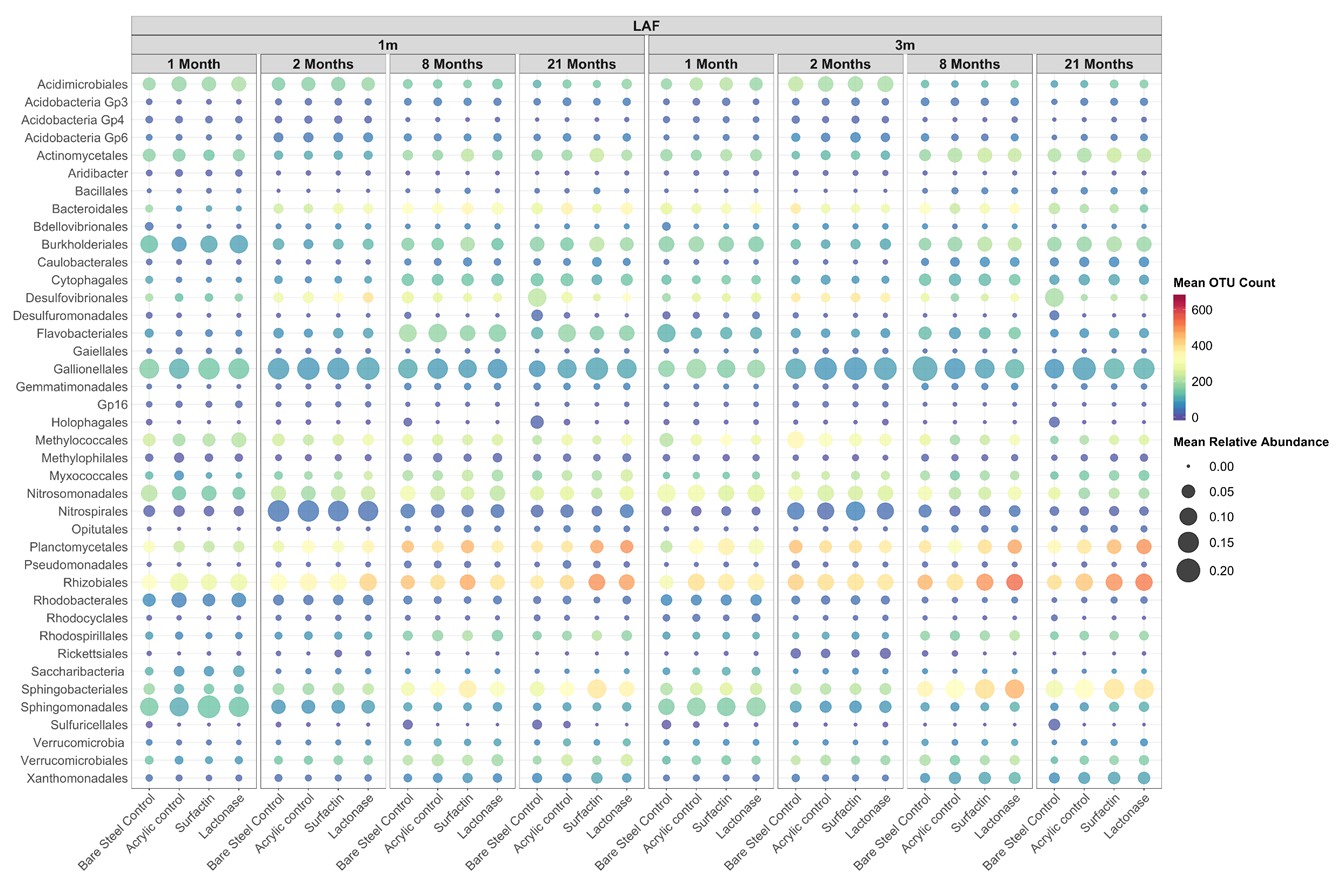
Fig S10**. Heatmap comparing the relative abundance of partial 16S rRNA sequences and OTU richness for the top 40 bacterial orders in all samples from each treatment and control. Diversity is indicated by the number of OTUs in each bacterial order.


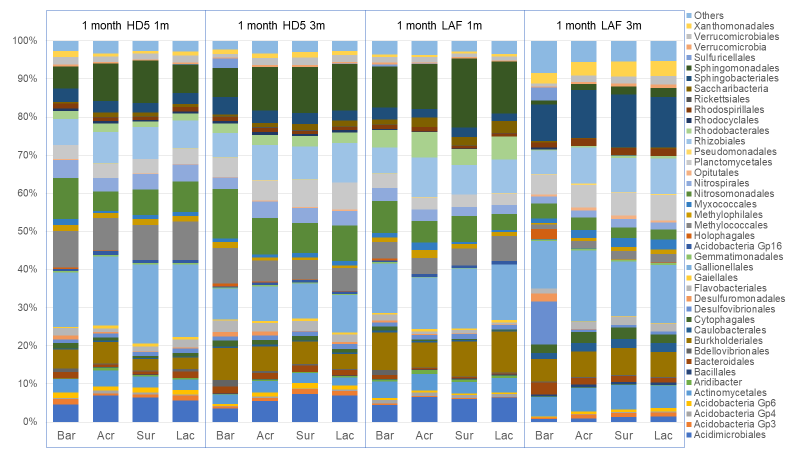


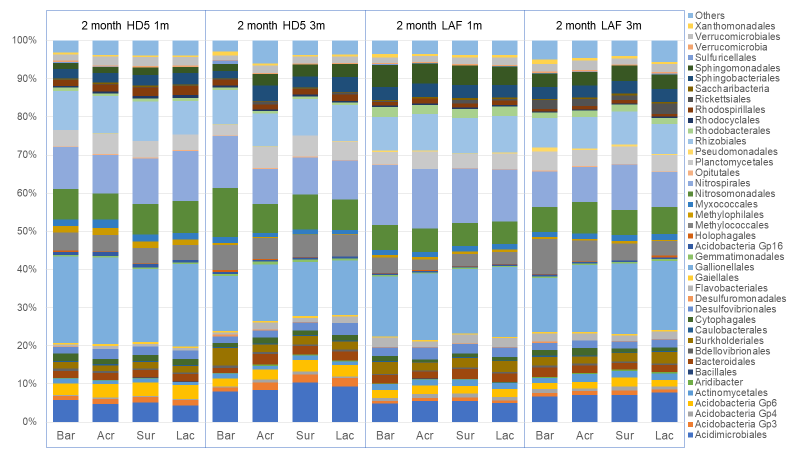


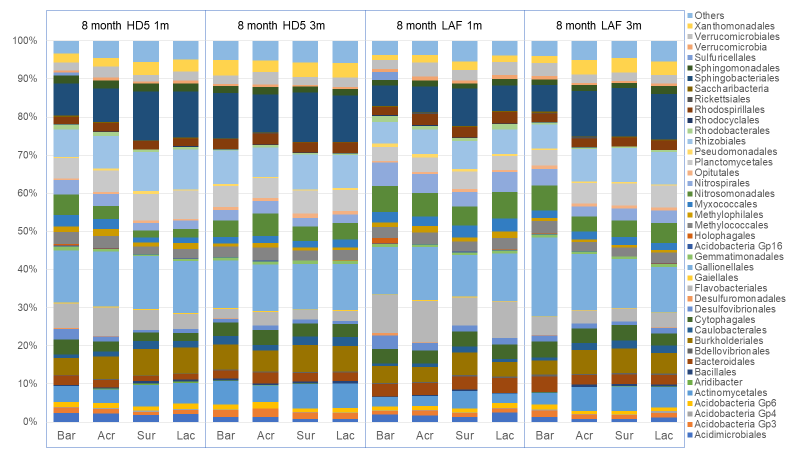


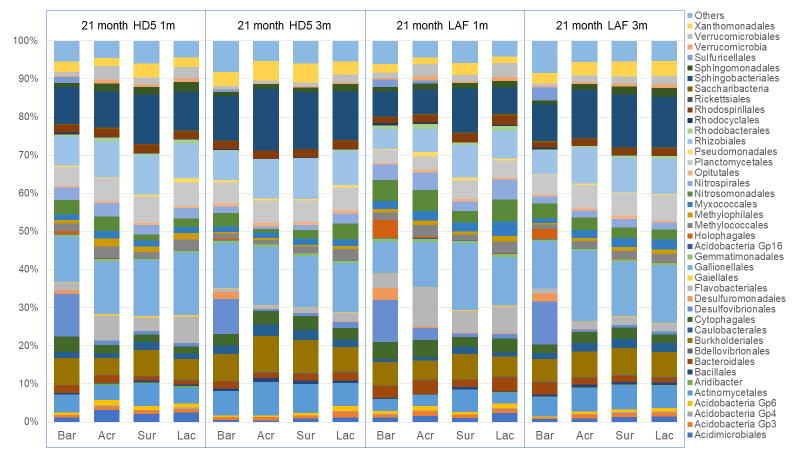


# **Fig S11**. Relative abundance of partial 16S rRNA sequences for the top 40 bacterial orders of all samples from each treatment and control. Bar: Bare steel control without coating, Acr: Acrylic coating control, Sur: Acrylic coating with 200 ug/ml surfactin, Lac: Acrylic coating with 200 ug/ml SsoPox lactonase enzyme.


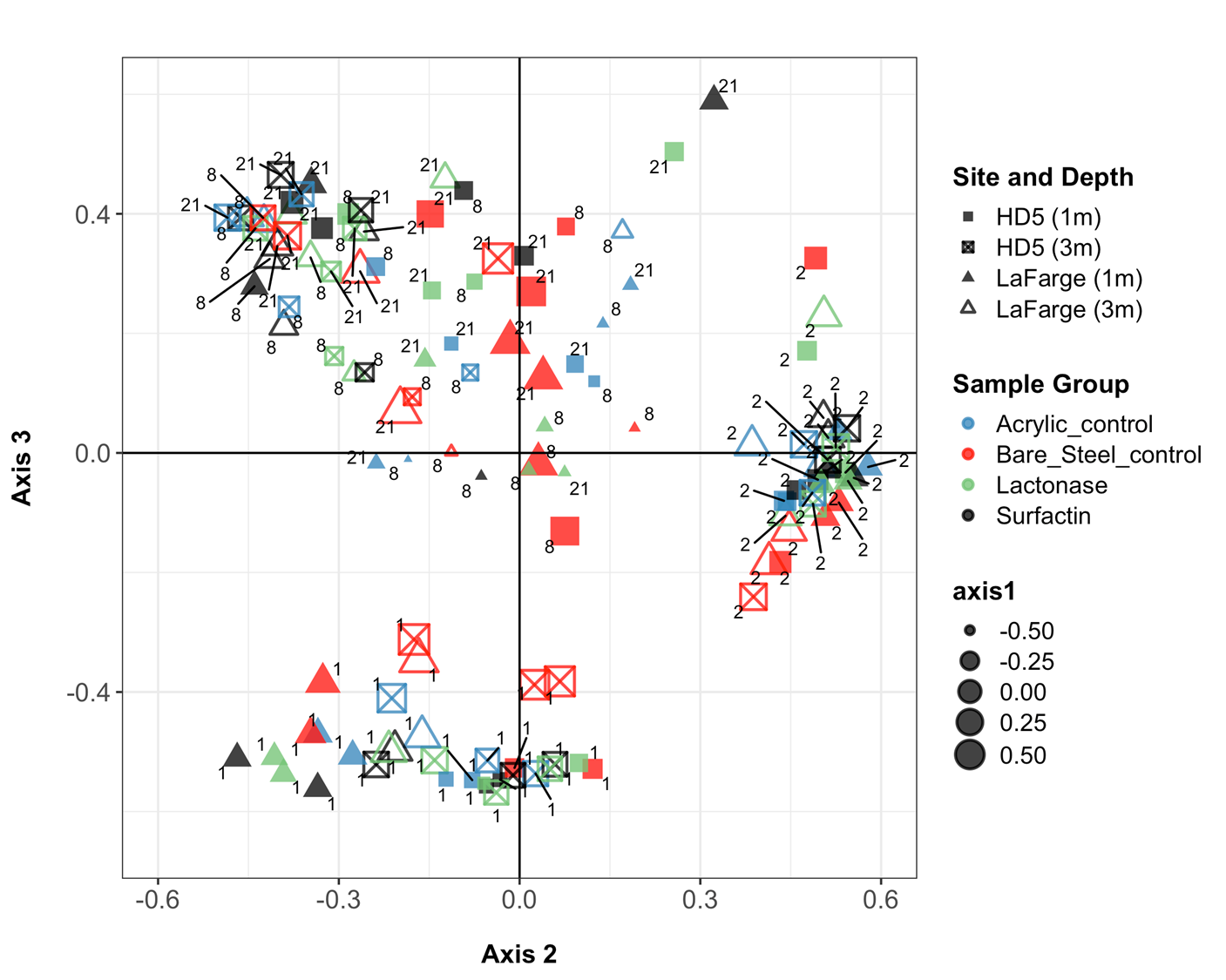


# **Fig S12**. Nonmetric multidimensional scaling plot showing the differences between bacterial communities in different treatments on corroding steel coupons grouped by site and sample exposure time. The sample time of each sample is shown on the graph by number markings. The 4 treatment groups are separated by different colors, and the sample depths are separated by filled and hollow shapes. The stress value of this NMDS plot is lower than 0.1.


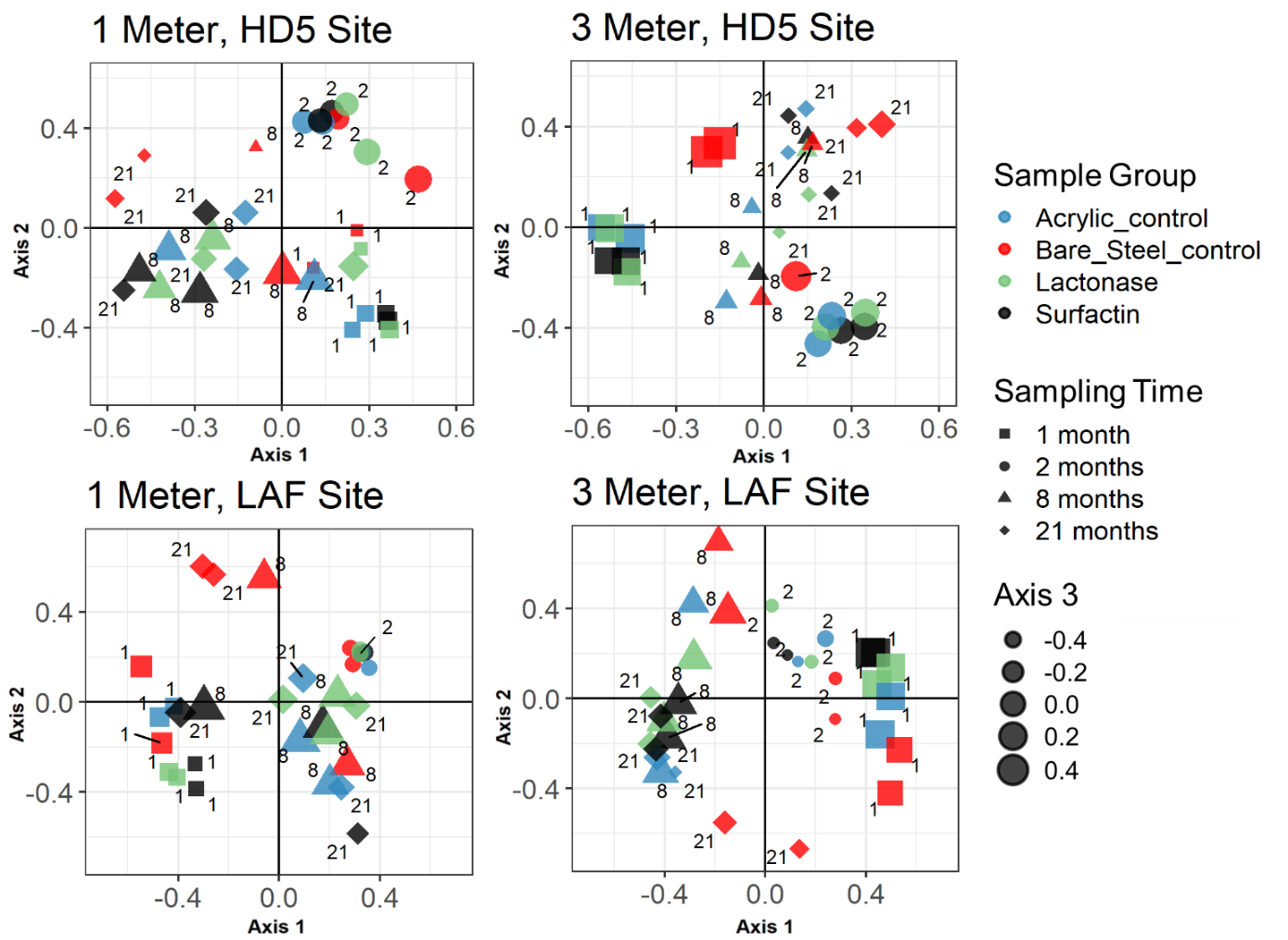


# **Fig S13**. Nonmetric multidimensional scaling plot showing the differences between bacterial communities in different treatments on corroding steel coupons over time. The site and depth of each plot is shown above the graph. The 4 treatment groups are separated by different colors, and the sample months are separated by shapes. The stress value of each NMDS plot is lower than 0.1. NMDS plots of all data combined and of each site, depth and month are shown in Fig S10.


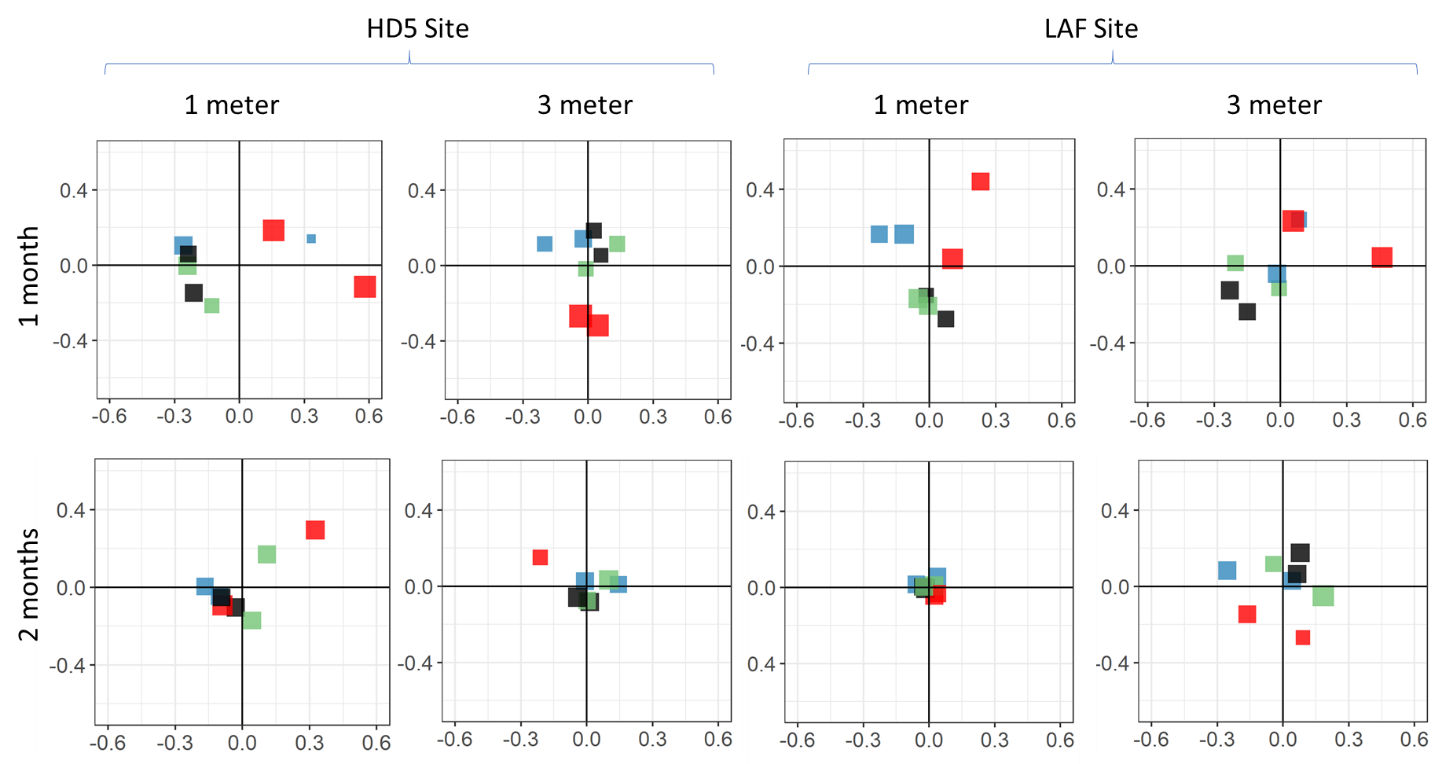


**Axis 2**


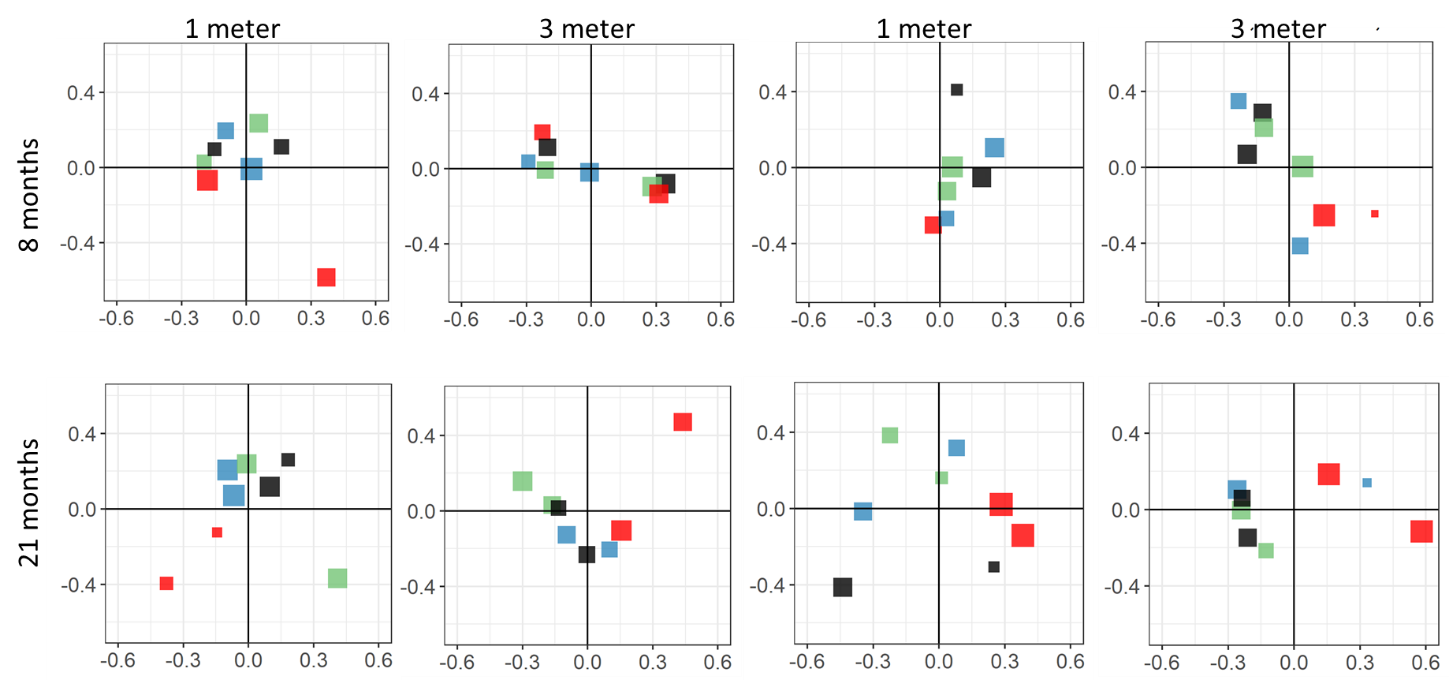


**Axis 1**


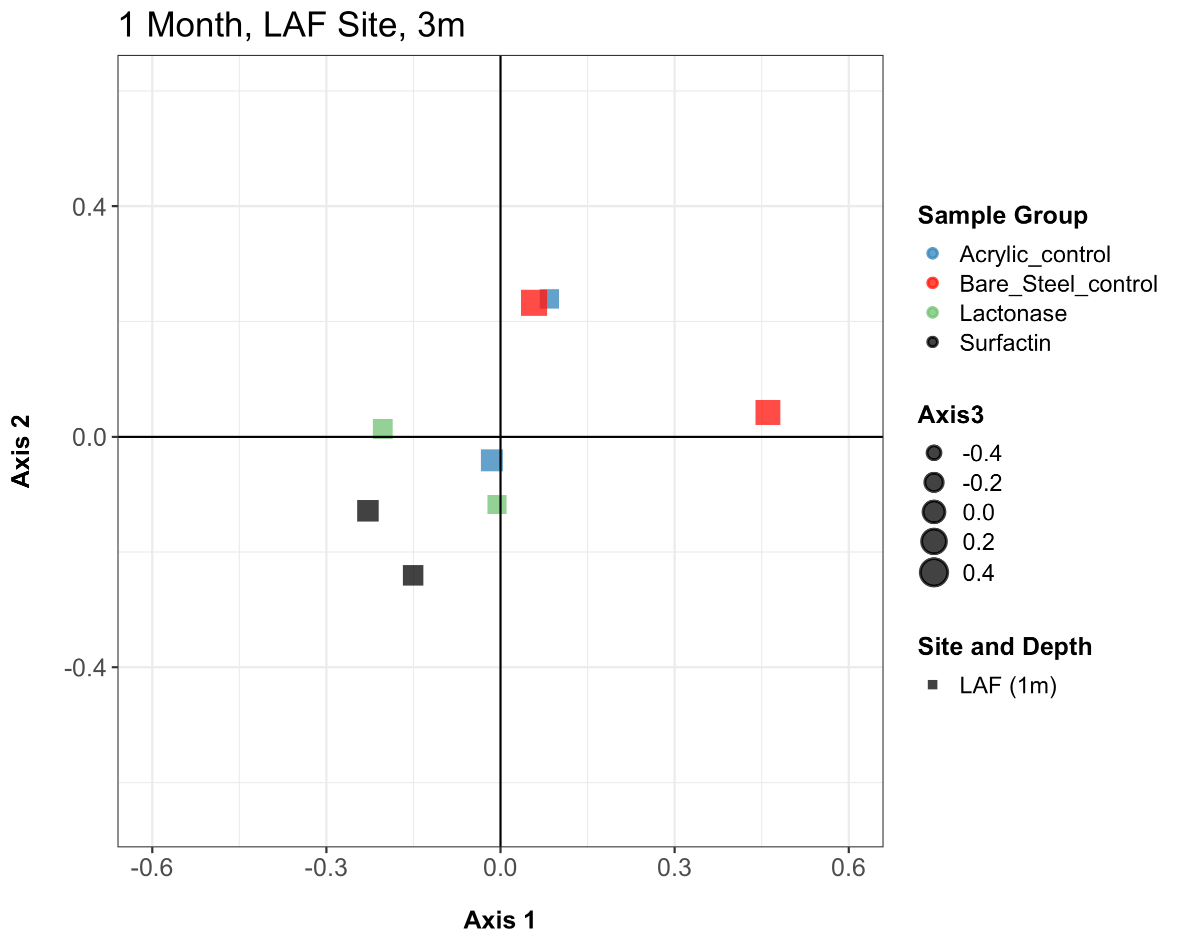

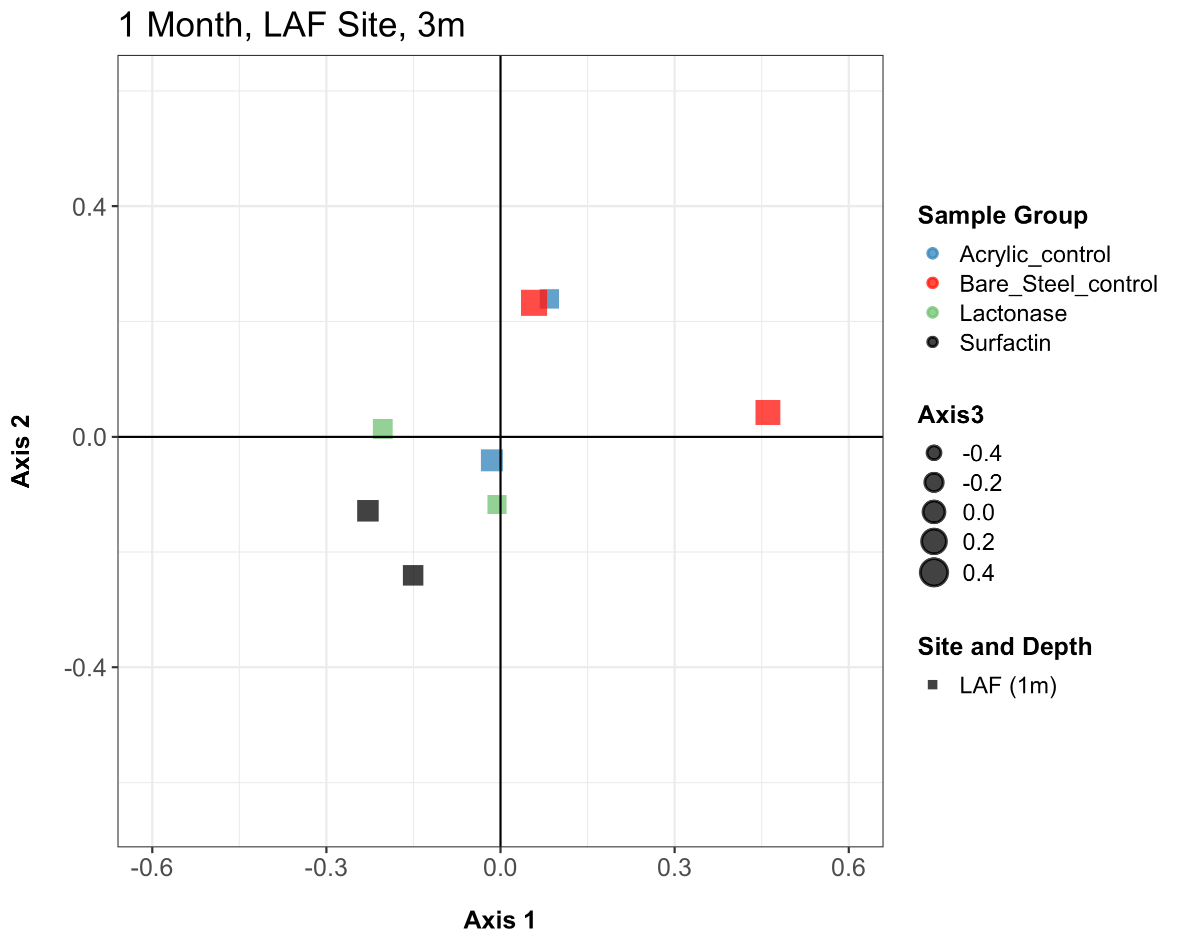


# **Fig S14**. Nonmetric multidimensional scaling plot showing the differences between bacterial communities in different treatments on corroding steel coupons grouped by site and sample exposure time. The site, depth and months of each plot is shown above the graph. The 4 treatment groups are separated by different colors. The stress value of each NMDS plot is lower than 0.2.


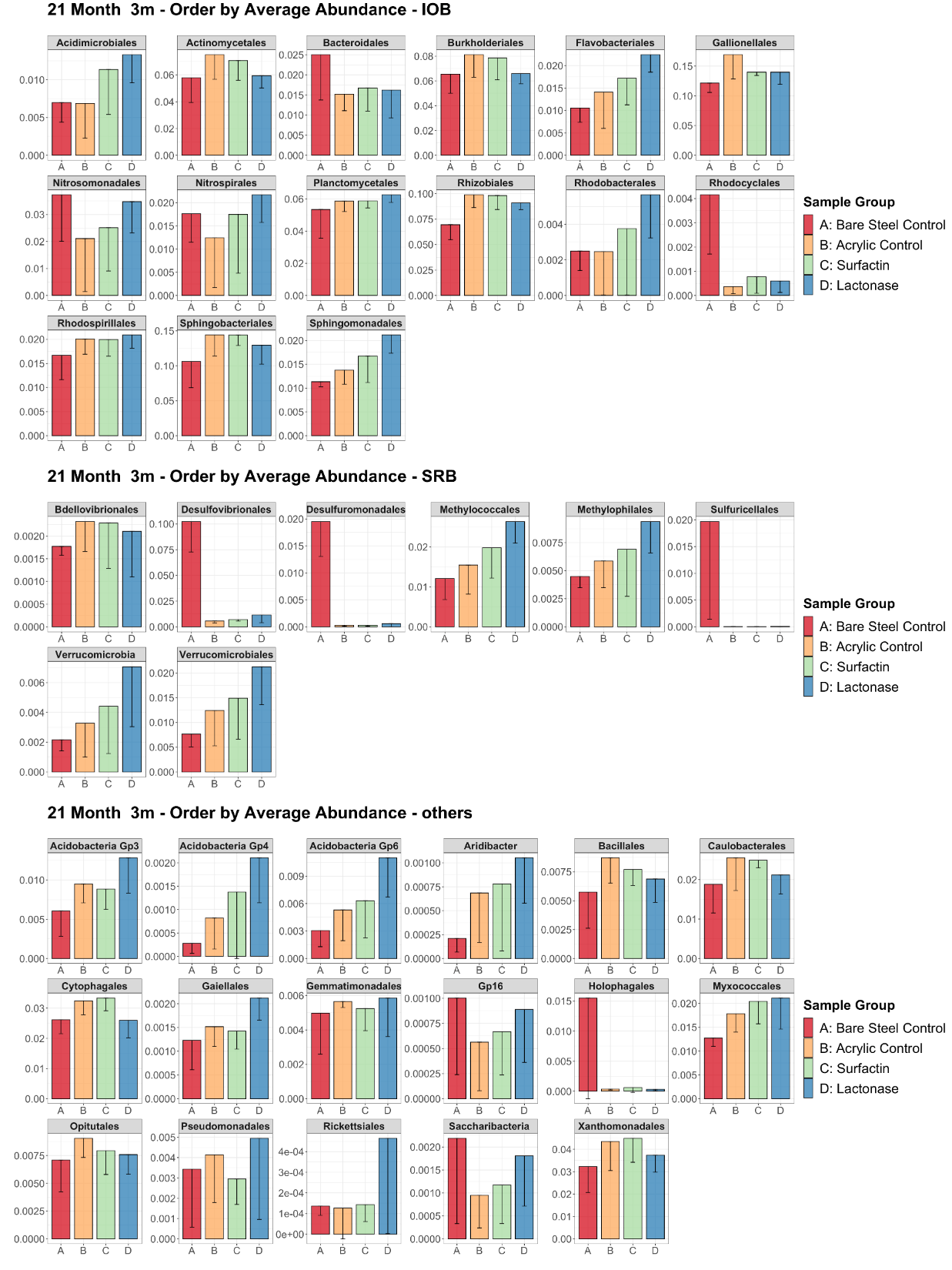


# **Fig S15**. Average iron-oxidizing and sulfate-reducing bacteria relative abundances on steel coupons with different experimental treatments. A: Bare steel control without coating, B: Acrylic coating control, C: Acrylic coating with 200 ug/ml surfactin, D: Acrylic coating with 200 ug/ml *Sso*Pox lactonase enzyme. Mean values of 2 coupons of 21-month 3m at 2 sites are shown (n=4).


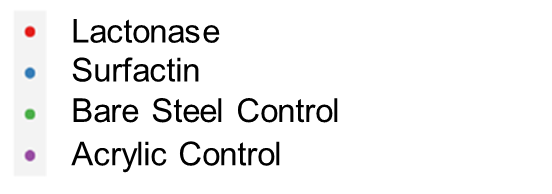


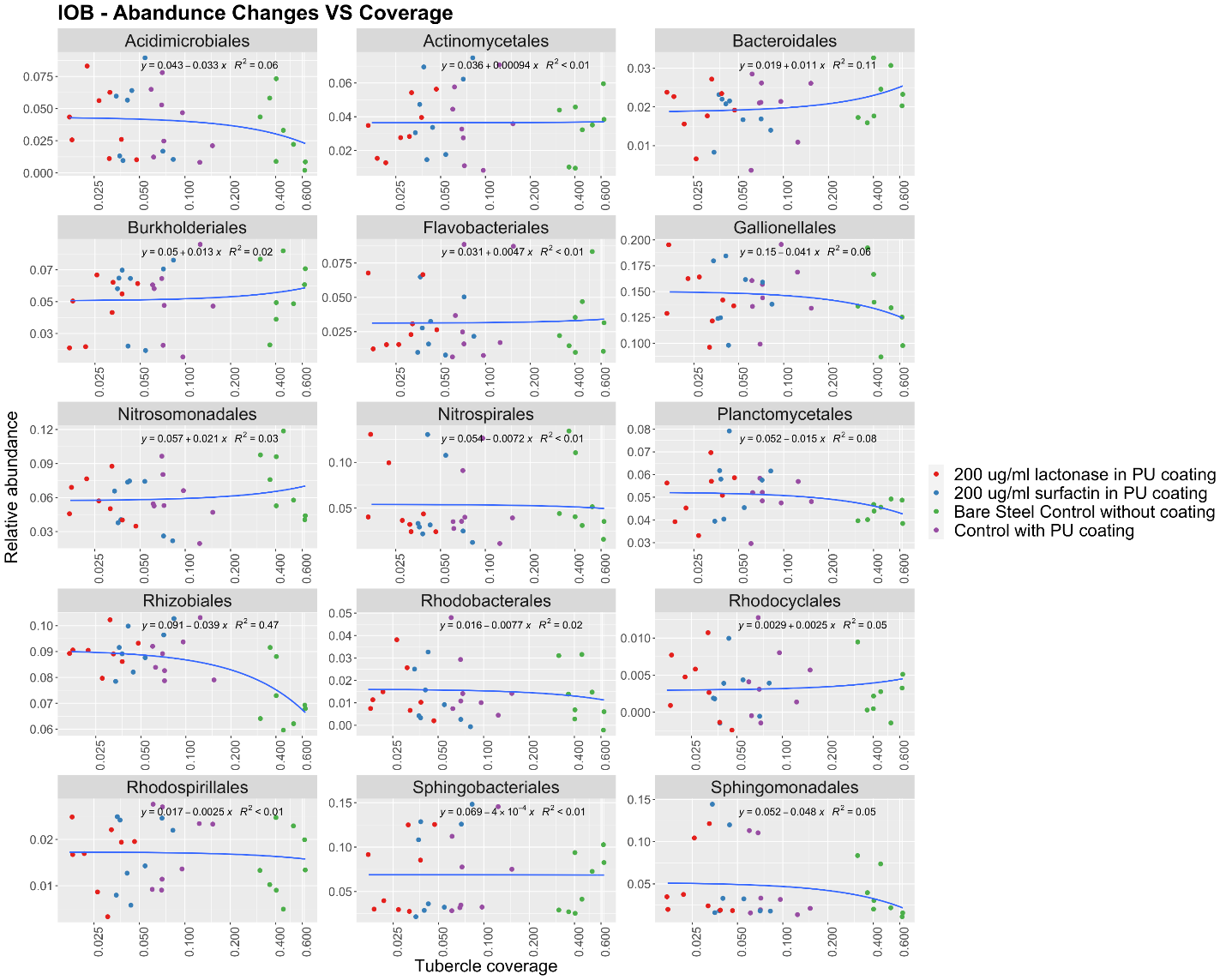


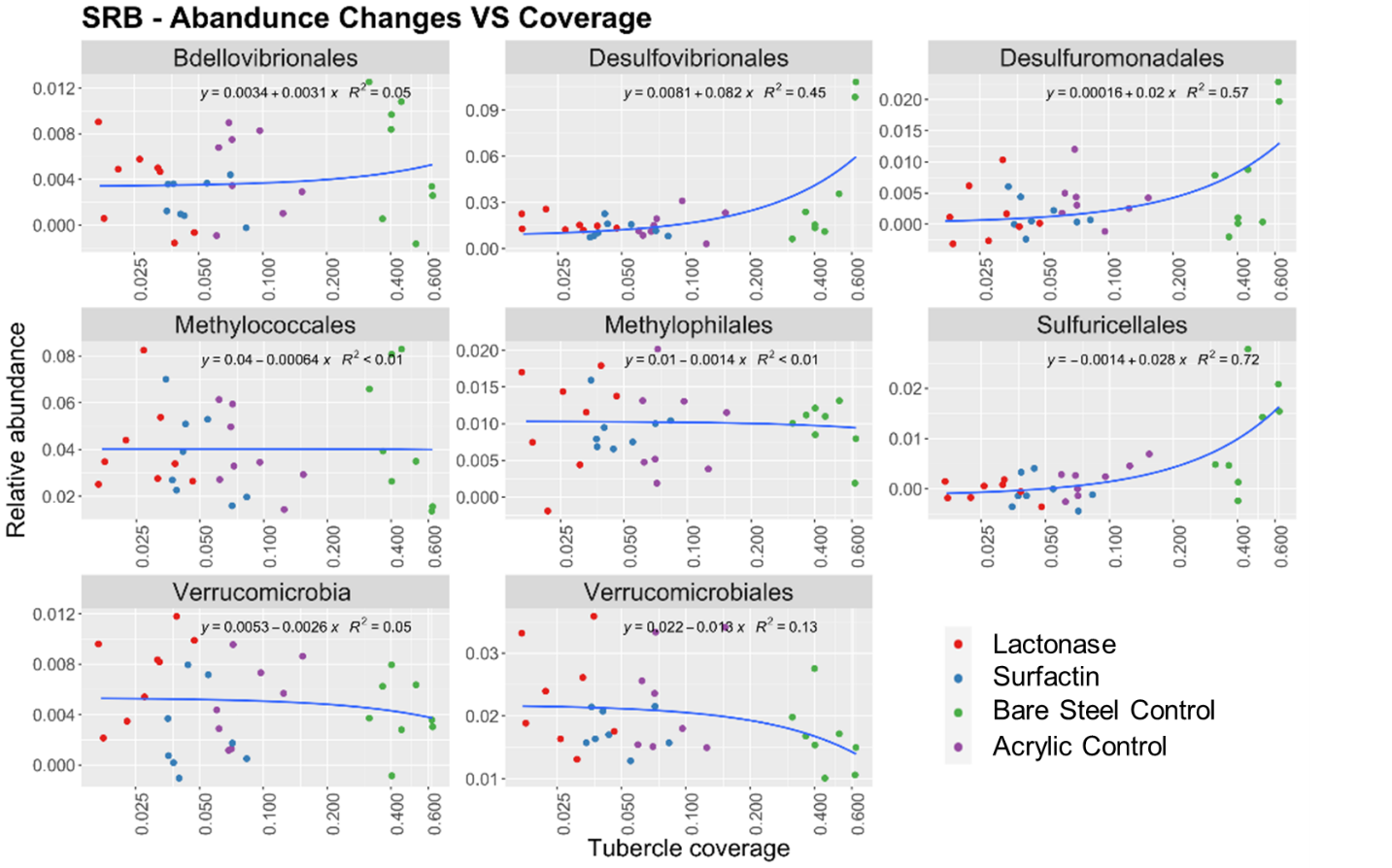


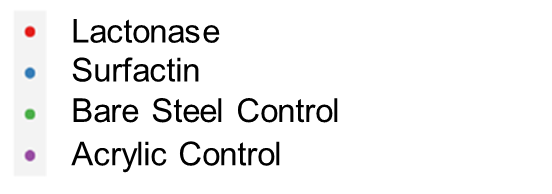

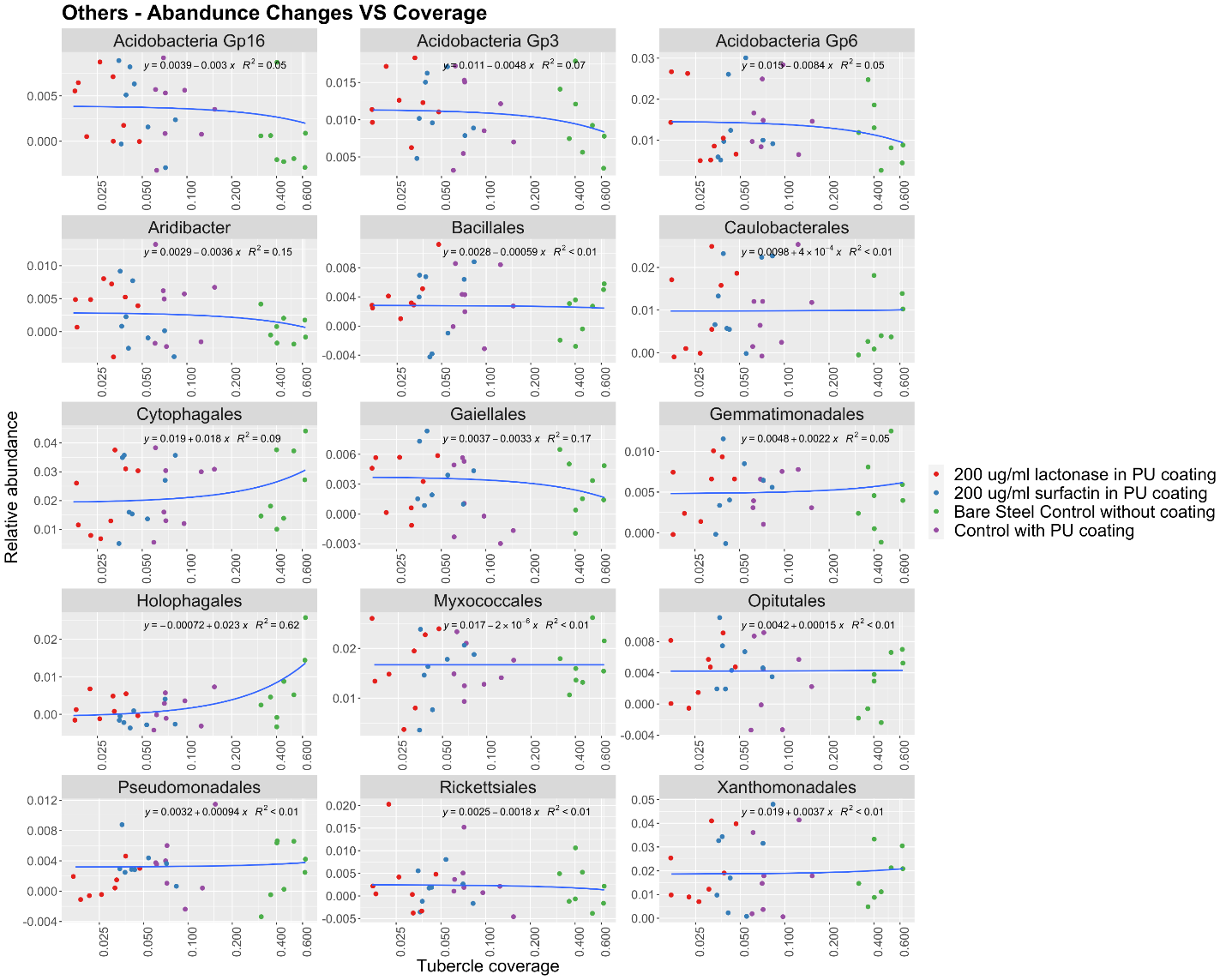


# **Fig S16**. Log scale plots showing the correlation between the relative abundance of bacterial orders and the tubercle coverage for the 40 most abundant bacterial orders by total sequence counts for treatment and control samples. The bacterial orders are grouped by IOB, SRB and others. All sites and depths are combined in the analysis.


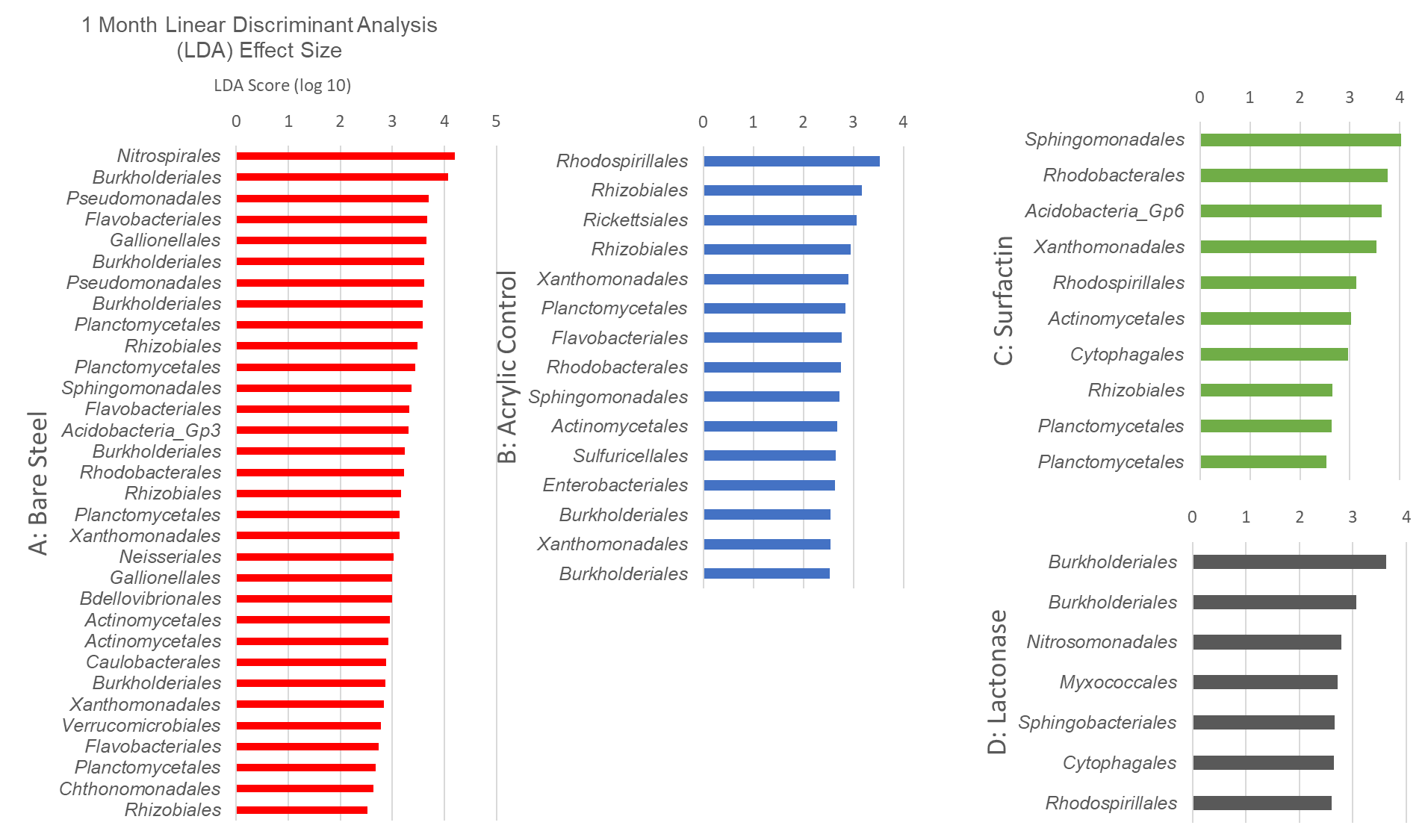

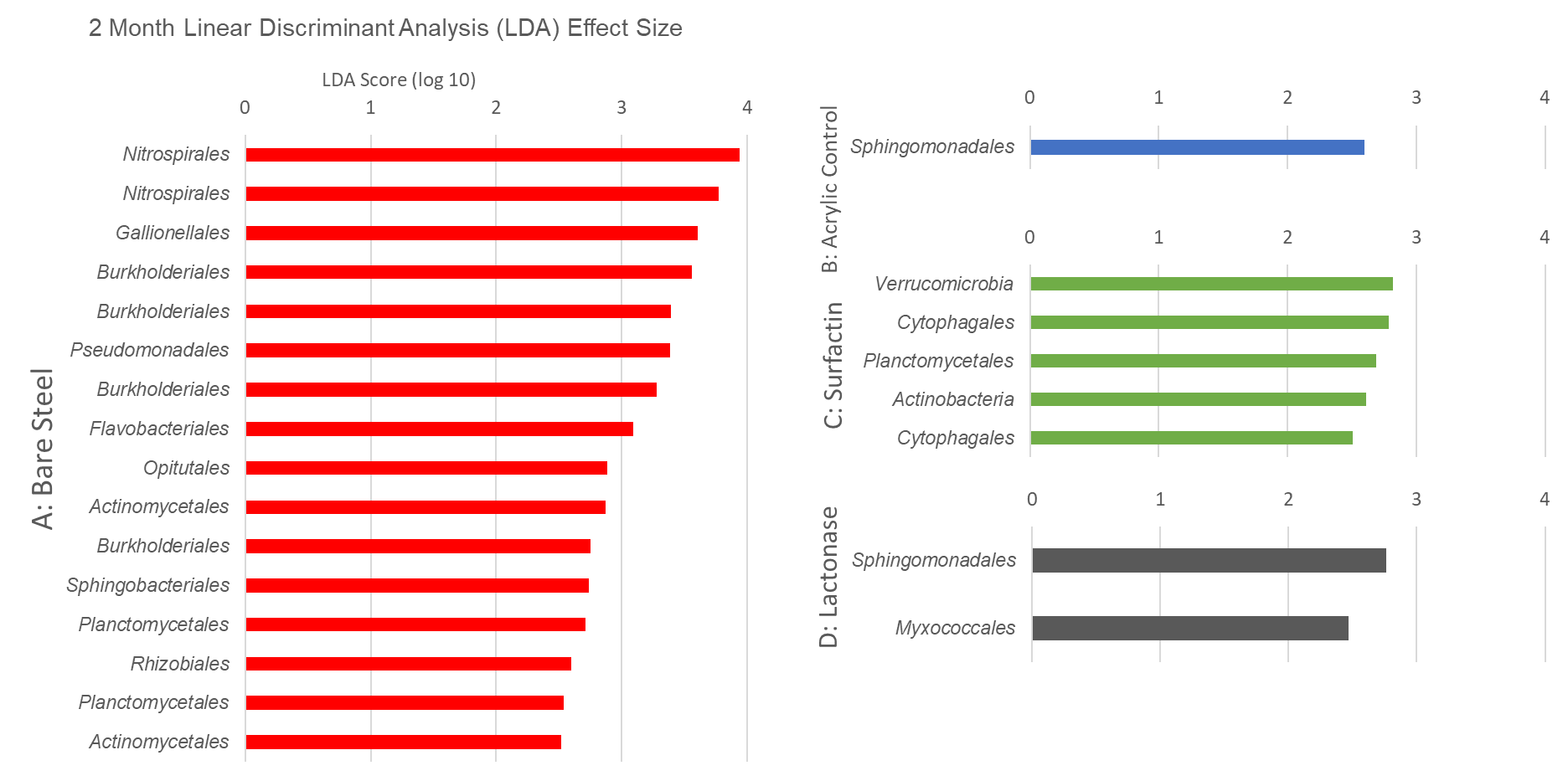


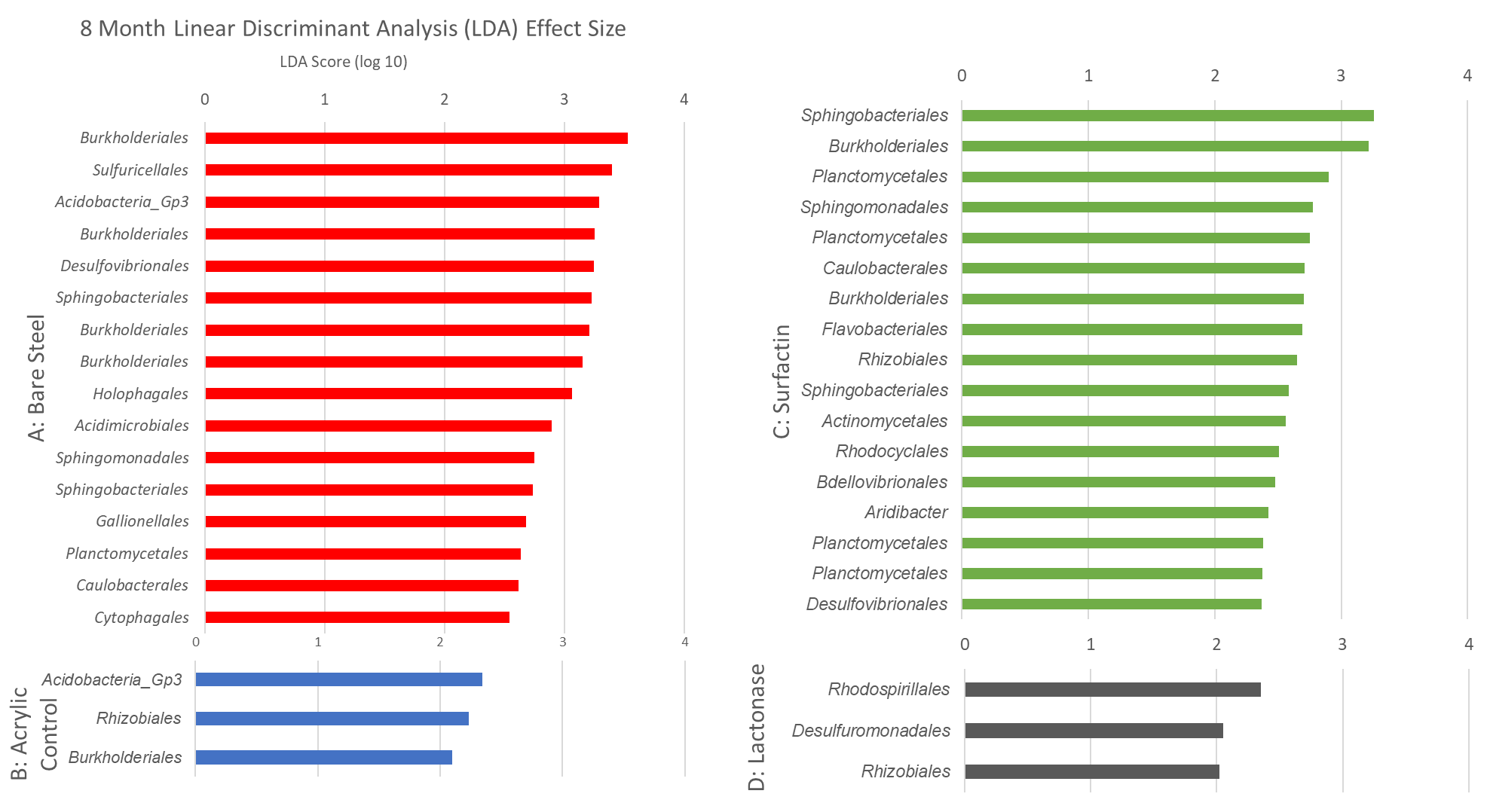


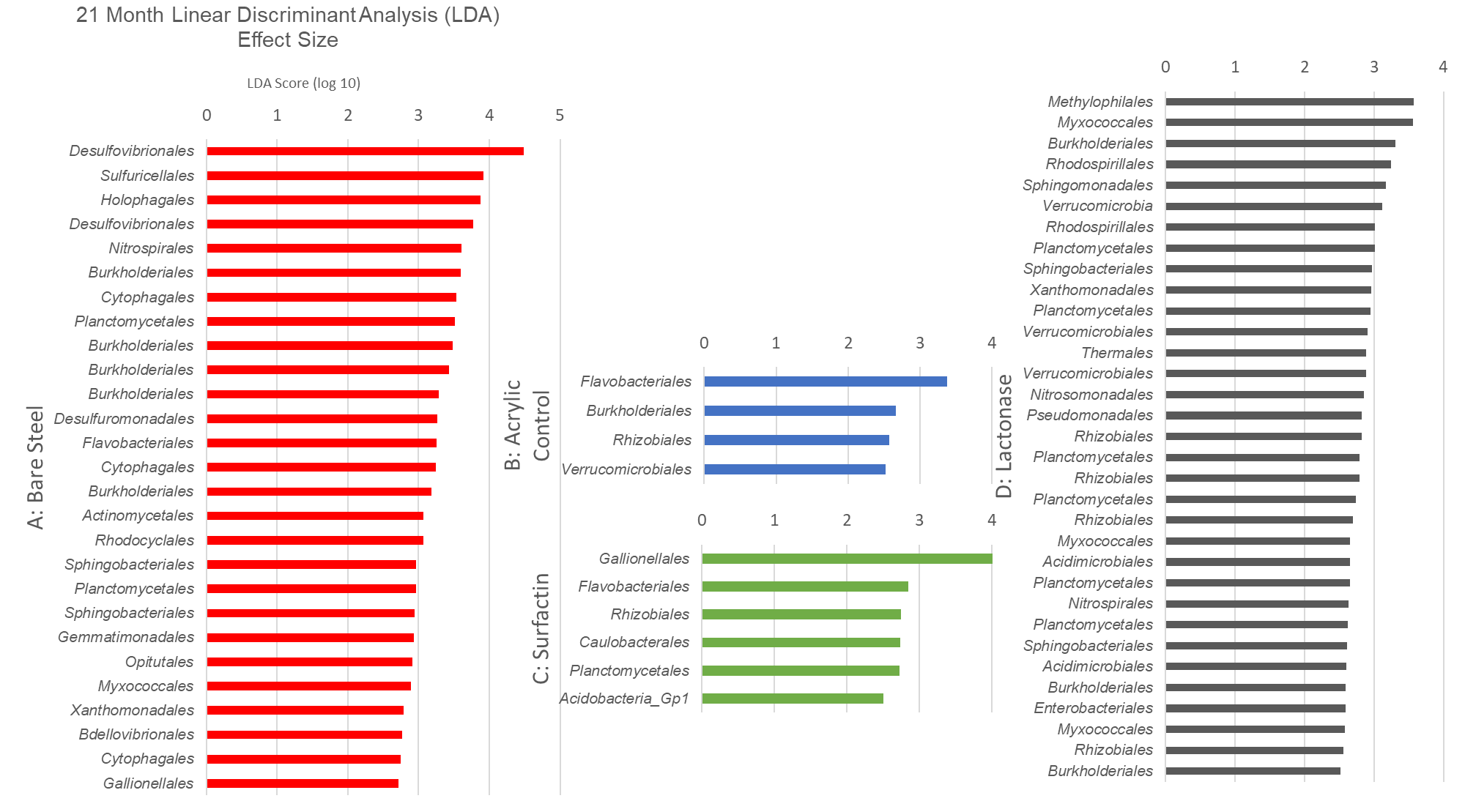


# **Fig S17**. The linear discriminant analysis (LDA) effect size (LEfSe) analysis identified bacterial orders that responded significantly to the different treatments during the four sampling periods. Each bar represents the effect size of an OTU. All sites and depths were combined in this analysis. Relative abundance was significant when P < 0.05, logarithmic LDA score ≥2.

# References for Table S2:

1. Dwidar M, Jang H, Sangwan N, Mun W, Im H, Yoon S, Choi S, Nam D, Mitchell RJ. Diffusible signaling factor, a quorum-sensing molecule, interferes with and is toxic towards Bdellovibrio bacteriovorus 109J. Microbial Ecology. 2021 Feb;81(2):347-56.
2. Sivakumar K, Scarascia G, Zaouri N, Wang T, Kaksonen AH, Hong PY. Salinity-mediated increment in sulfate reduction, biofilm formation, and quorum sensing: a potential connection between quorum sensing and sulfate reduction?. Frontiers in microbiology. 2019 Feb 6;10:188.
3. Scarascia G, Wang T, Hong PY. Quorum sensing and the use of quorum quenchers as natural biocides to inhibit sulfate-reducing bacteria. Antibiotics. 2016 Dec;5(4):39.
4. Ayangbenro AS, Olanrewaju OS, Babalola OO. Sulfate-reducing bacteria as an effective tool for sustainable acid mine bioremediation. Frontiers in microbiology. 2018 Aug 22;9:1986.
5. Ranava D, Backes C, Karthikeyan G, Ouari O, Soric A, Guiral M, Cárdenas ML, Giudici-Orticoni MT. Metabolic exchange and energetic coupling between nutritionally stressed bacterial species: role of quorum-sensing molecules. Mbio. 2021 Jan 19;12(1):e02758-20.
6. Scarascia G, Lehmann R, Machuca LL, Morris C, Cheng KY, Kaksonen A, Hong PY. Effect of quorum sensing on the ability of Desulfovibrio vulgaris to form biofilms and to biocorrode carbon steel in saline conditions. Applied and environmental microbiology. 2019 Dec 13;86(1):e01664-19.
7. Enning D, Garrelfs J. Corrosion of iron by sulfate-reducing bacteria: new views of an old problem. Applied and environmental microbiology. 2014 Feb 15;80(4):1226-36.
8. Badalamenti JP, Summers ZM, Chan CH, Gralnick JA, Bond DR. Isolation and genomic characterization of ‘Desulfuromonas soudanensis WTL’, a metal-and electrode-respiring bacterium from anoxic deep subsurface brine. Frontiers in microbiology. 2016 Jun 21;7:913.
9. Puri AW, Schaefer AL, Fu Y, Beck DA, Greenberg EP, Lidstrom ME. Quorum sensing in a methane-oxidizing bacterium. Journal of bacteriology. 2017 Feb 14;199(5):e00773-16.
10. Su Y, Yang Y, Zhu XY, Zhang XH, Yu M. Metagenomic insights into the microbial assemblage capable of quorum sensing and quorum quenching in particulate organic matter in the Yellow Sea. Frontiers in microbiology. 2020;11.
11. Zhou Q, Xie X, Feng F, Huang S, Sun Y. Impact of acyl-homoserine lactones on the response of nitrogen cycling in sediment to florfenicol stress. Science of The Total Environment. 2021 Sep 1;785:147294.
12. Panchavinin S, Tobino T, Hara-Yamamura H, Matsuura N, Honda R. Candidates of quorum sensing bacteria in activated sludge associated with N-acyl homoserine lactones. Chemosphere. 2019 Dec 1;236:124292.
13. Mulya E, Waturangi DE. Screening and quantification of anti-quorum sensing and antibiofilm activity of Actinomycetes isolates against food spoilage biofilm-forming bacteria. BMC microbiology. 2021 Dec;21(1):1-8.
14. Polkade AV, Mantri SS, Patwekar UJ, Jangid K. Quorum sensing: an under-explored phenomenon in the phylum Actinobacteria. Frontiers in microbiology. 2016 Feb 10;7:131.
15. Oliveira RA, Cabral V, Torcato I, Xavier KB. Deciphering the quorum-sensing lexicon of the gut microbiota. Cell Host & Microbe. 2023 Apr 12;31(4):500-12.
16. Tan CH, Koh KS, Xie C, Tay M, Zhou Y, Williams R, Ng WJ, Rice SA, Kjelleberg S. The role of quorum sensing signalling in EPS production and the assembly of a sludge community into aerobic granules. The ISME journal. 2014 Jun;8(6):1186-97.
17. Zuniga A, Donoso RA, Ruiz D, Ruz GA, González B. Quorum-sensing systems in the plant growth-promoting bacterium Paraburkholderia phytofirmans PsJN exhibit cross-regulation and are involved in biofilm formation. Molecular Plant-Microbe Interactions. 2017 Jul 27;30(7):557-65.
18. Su Y, Tang K, Liu J, Wang Y, Zheng Y, Zhang XH. Quorum sensing system of Ruegeria mobilis Rm01 controls lipase and biofilm formation. Frontiers in microbiology. 2019 Jan 9;9:3304.
19. Kimura N. Metagenomic approaches to understanding phylogenetic diversity in quorum sensing. Virulence. 2014 Apr 1;5(3):433-42.
20. Burton EO, Read HW, Pellitteri MC, Hickey WJ. Identification of acyl-homoserine lactone signal molecules produced by Nitrosomonas europaea strain Schmidt. Applied and environmental microbiology. 2005 Aug;71(8):4906-9.
21. Wu J, Gao H, Ye J, Chang Y, Yu R, Ding Z, Zhu G. Effects of Exogenous N-Acyl-Homoserine Lactone as Signal Molecule on Nitrosomonas Europaea under ZnO Nanoparticle Stress. International journal of environmental research and public health. 2019 Jan;16(16):3003.
22. Geets J, Boon N, Verstraete W. Strategies of aerobic ammonia-oxidizing bacteria for coping with nutrient and oxygen fluctuations. FEMS microbiology ecology. 2006 Oct 1;58(1):1-3.
23. Kalia VC, Raju SC, Purohit HJ. Genomic analysis reveals versatile organisms for quorum quenching enzymes: acyl-homoserine lactone-acylase and-lactonase. The open microbiology journal. 2011;5:1.
24. Graça AP, Calisto R, Lage OM. Planctomycetes as novel source of bioactive molecules. Frontiers in microbiology. 2016 Aug 12;7:1241.
25. Collins AJ, Fullmer MS, Gogarten JP, Nyholm SV. Comparative genomics of Roseobacter clade bacteria isolated from the accessory nidamental gland of Euprymna scolopes. Frontiers in microbiology. 2015 Feb 23;6:123.
26. Li Q, Xu X, He C, Zheng L, Gao W, Sun C, Li J, Gao F. Complete Genome Sequence of a Quorum-Sensing Bacterium, Oceanicola sp. Strain D3, Isolated from a Microplastic Surface in Coastal Water of Qingdao, China. Microbiology resource announcements. 2019 Oct 3;8(40):e01022-19.
27. Britstein M, Devescovi G, Handley KM, Malik A, Haber M, Saurav K, Teta R, Costantino V, Burgsdorf I, Gilbert JA, Sher N. A new N-Acyl homoserine lactone synthase in an uncultured symbiont of the Red Sea sponge Theonella swinhoei. Applied and environmental microbiology. 2016 Feb 15;82(4):1274-85.
28. Ryu DH, Lee SW, Mikolaityte V, Kim YW, Jeong HY, Lee SJ, Lee CH, Lee JK. Identification of a Second Type of AHL-lactonase from Rhodococcus sp. BH4, belonging to the α/β Hydrolase Superfamily. J Microbiol Biotechnol. 2020 Jun 28;30(6):937-945. doi: 10.4014/jmb.2001.01006.
29. Vial L, Cuny C, Gluchoff-Fiasson K, Comte G, Oger PM, Faure D, Dessaux Y, Bally R, Wisniewski-Dyé F. N-acyl-homoserine lactone-mediated quorum-sensing in Azospirillum: an exception rather than a rule. FEMS microbiology ecology. 2006 Nov 1;58(2):155-68.
30. Zhang JW, Xuan CG, Lu CH, Guo S, Yu JF, Asif M, Jiang WJ, Zhou ZG, Luo ZQ, Zhang LQ. AidB, a novel thermostable N-acylhomoserine lactonase from the bacterium Bosea sp. Applied and environmental microbiology. 2019 Nov 27;85(24):e02065-19.
31. Gram L, Grossart HP, Schlingloff A, Kiørboe T. Possible quorum sensing in marine snow bacteria: production of acylated homoserine lactones by Roseobacter strains isolated from marine snow. Applied and Environmental Microbiology. 2002 Aug;68(8):4111-6.
32. Huang X, Zhu J, Cai Z, Lao Y, Jin H, Yu K, Zhang B, Zhou J. Profiles of quorum sensing (QS)-related sequences in phycospheric microorganisms during a marine dinoflagellate bloom, as determined by a metagenomic approach. Microbiological research. 2018 Dec 1;217:1-3.
33. Morohoshi T, Kamimura Y, Sato N, Iizumi T. Distribution and characterization of N-acylhomoserine lactone (AHL)-degrading activity and AHL lactonase gene (qsdS) in Sphingopyxis. Journal of bioscience and bioengineering. 2019 Apr 1;127(4):411-7.
34. Gan HM, Gan HY, Ahmad NH, Aziz NA, Hudson AO, Savka MA. Whole genome sequencing and analysis reveal insights into the genetic structure, diversity and evolutionary relatedness of luxI and luxR homologs in bacteria belonging to the Sphingomonadaceae family. Frontiers in cellular and infection microbiology. 2015 Jan 8;4:188.
35. Kalam S, Basu A, Ahmad I, Sayyed RZ, El Enshasy HA, Dailin DJ, Suriani N. Recent understanding of soil Acidobacteria and their ecological significance: A critical review. Frontiers in Microbiology. 2020;11:2712.
36. Biswa P, Doble M. Production of acylated homoserine lactone by Gram-positive bacteria isolated from marine water. FEMS microbiology letters. 2013 Jun 1;343(1):34-41.
37. See-Too WS, Ee R, Lim YL, Convey P, Pearce DA, Yin WF, Chan KG. AidP, a novel N-Acyl homoserine lactonase gene from Antarctic Planococcus sp. Scientific reports. 2017 Feb 22;7(1):1-1.
38. Biswa P, Doble M. Production of acylated homoserine lactone by Gram-positive bacteria isolated from marine water. FEMS microbiology letters. 2013 Jun 1;343(1):34-41.
39. Ma ZP, Lao YM, Jin H, Lin GH, Cai ZH, Zhou J. Diverse profiles of AI-1 type quorum sensing molecules in cultivable bacteria from the Mangrove (Kandelia obovata) rhizosphere environment. Frontiers in microbiology. 2016 Dec 5;7:1957.
40. Whitworth DE, Zwarycz A. A genomic survey of signalling in the myxococcaceae. Microorganisms. 2020 Nov;8(11):1739.
41. Albataineh H, Duke M, Misra SK, Sharp JS, Stevens DC. Identification of a solo acylhomoserine lactone synthase from the myxobacterium Archangium gephyra. Scientific reports. 2021 Feb 4;11(1):1-0.
42. Niu C, Clemmer KM, Bonomo RA, Rather PN. Isolation and characterization of an autoinducer synthase from Acinetobacter baumannii. Journal of bacteriology. 2008 May 1;190(9):3386-92.
43. Cai W, Zhang Z, Ren G, Shen Q, Hou Y, Ma A, Deng Y, Wang A, Liu W. Quorum sensing alters the microbial community of electrode-respiring bacteria and hydrogen scavengers toward improving hydrogen yield in microbial electrolysis cells. Applied Energy. 2016 Dec 1;183:1133-41.
44. Liébana R, Arregui L, Santos A, Murciano A, Marquina D, Serrano S. Unravelling the interactions among microbial populations found in activated sludge during biofilm formation. FEMS Microbiology Ecology. 2016 Sep 1;92(9).
45. Hidayanti, A. K., Gazali, A., & Tagami, Y. (2022). Effect of Quorum Sensing Inducers and Inhibitors on Cytoplasmic Incompatibility Induced by Wolbachia (Rickettsiales: Anaplasmataceae) in American Serpentine Leafminer (Diptera: Agromyzidae): Potential Tool for the Incompatible Insect Technique. Journal of Insect Science, 22(1), 8.
46. Huedo P, Coves X, Daura X, Gibert I, Yero D. Quorum sensing signaling and quenching in the multidrug-resistant pathogen Stenotrophomonas maltophilia. Frontiers in cellular and infection microbiology. 2018 Apr 24;8:122.
47. Ling J, Zhou L, Wu G, Zhao Y, Jiang T, Liu F. The AHL quorum-sensing system negatively regulates growth and autolysis in Lysobacter brunescens. Frontiers in microbiology. 2019 Dec 3;10:2748.
48. Korenblum E, de Araujo LV, Guimarães CR, De Souza LM, Sassaki G, Abreu F, Nitschke M, Lins U, Freire DM, Barreto-Bergter E, Seldin L. Purification and characterization of a surfactin-like molecule produced by Bacillus sp. H2O-1 and its antagonistic effect against sulfate reducing bacteria. BMC microbiology. 2012 Dec;12(1):1-3.
49. Chunxiao D, Ma F, Wu W, Li S, Yang J, Chen Z, Lian S, Qu Y. Metagenomic analysis reveals indole signaling effect on microbial community in sequencing batch reactors: Quorum sensing inhibition and antibiotic resistance enrichment. Environmental Research. 2023 Jul 15;229:115897.
